# Molecular Characteristics and Pathogenicity of *Staphylococcus aureus* Exotoxins

**DOI:** 10.3390/ijms25010395

**Published:** 2023-12-28

**Authors:** Zhihao Zhu, Zuo Hu, Shaowen Li, Rendong Fang, Hisaya K. Ono, Dong-Liang Hu

**Affiliations:** 1 Department of Zoonoses, Kitasato University School of Veterinary Medicine, Towada 034-8628, Japan; zhuzhihao@webmail.hzau.edu.cn (Z.Z.); pegplus12138jp@gmail.com (Z.H.); hisaono@vmas.kitasato-u.ac.jp (H.K.O.); 2 College of Veterinary Medicine, Huazhong Agricultural University, Wuhan 430070, China; lishaowen@mail.hzau.edu.cn; 3 Joint International Research Laboratory of Animal Health and Animal Food Safety, College of Veterinary Medicine, Southwest University, Chongqing 400715, China; rdfang@swu.edu.cn

**Keywords:** staphylococcal enterotoxin, superantigen, membrane-damaging toxin, hemolysin, Panton–Valentine leucocidin, exfoliative toxin

## Abstract

*Staphylococcus aureus* stands as one of the most pervasive pathogens given its morbidity and mortality worldwide due to its roles as an infectious agent that causes a wide variety of diseases ranging from moderately severe skin infections to fatal pneumonia and sepsis. *S. aureus* produces a variety of exotoxins that serve as important virulence factors in *S. aureus*-related infectious diseases and food poisoning in both humans and animals. For example, staphylococcal enterotoxins (SEs) produced by *S. aureus* induce staphylococcal foodborne poisoning; toxic shock syndrome toxin-1 (TSST-1), as a typical superantigen, induces toxic shock syndrome; hemolysins induce cell damage in erythrocytes and leukocytes; and exfoliative toxin induces staphylococcal skin scalded syndrome. Recently, Panton–Valentine leucocidin, a cytotoxin produced by community-associated methicillin-resistant *S. aureus* (CA-MRSA), has been reported, and new types of SEs and staphylococcal enterotoxin-like toxins (SEls) were discovered and reported successively. This review addresses the progress of and novel insights into the molecular structure, biological activities, and pathogenicity of both the classic and the newly identified exotoxins produced by *S. aureus*.

## 1. Introduction

*Staphylococcus aureus* infection results in intractable infectious diseases, establishing a severe and persistent pathology within the host by circumventing host defense mechanisms. *S. aureus* has become a serious problem in human hospital infections. It is also associated with important pathology in veterinary medicine. In particular, *S. aureus* and the produced exotoxins can cause a variety of diseases in mammals and birds, such as infectious mastitis in cattle, exfoliative dermatitis in pigs, pyoderma in dogs, and edematous dermatitis/arthritis in poultry [1,2]. The economic ramifications of *S. aureus* infection extend beyond human health care, inflicting substantial losses in dairy and livestock industries. The surging of methicillin-resistant *S. aureus* (MRSA) compounds represents an important issue and is becoming a major problem in medical and veterinary medicine. MRSA was first reported in the early 1960s, and MRSA infection rates increased dramatically in the late 1970s, mainly in hospitalized patients (health care-associated MRSA, HA-MRSA). In the 1990s, community-acquired MRSA (CA-MRSA) infections in previously healthy individuals were recognized. Furthermore, multi-drug-resistant MRSA strains have continued to emerge and increase, causing concern in the medical and veterinary medical systems. However, there is currently a lack of effective protective measures and preventive vaccines. Previous studies have reported that vaccines using inactivated bacterial cells, capsules, and toxins as immunogens have shown some or no effect and have not yet been applied in the clinical stage. Some studies have shown that the use of superantigen (SAg) exotoxins as immunogens has a preventive effect on the establishment and persistence of *S. aureus* infection in animal models [3,4]. There is an urgent need to clarify the pathogenic mechanism of this bacterium and develop an effective vaccine based on the infection-immunity mechanism between the bacteria and hosts.

*S. aureus* produces an array of exotoxins and proteases that contribute to their ability to colonize and cause disease in humans and animals [1,5]. The primary exotoxins of *S. aureus* fall into three major categories: superantigen toxins, membrane-damaged toxins (MDTs), and exfoliative toxins (ETs). The SAg toxins include staphylococcal enterotoxins (SEs), which have emetic activity that induce staphylococcal food poisoning, and toxic shock syndrome toxin-1 (TSST-1), which is a typical superantigen that induces toxic shock syndrome (TSS) in humans. MDTs can be further divided into hemolysins; leukocidins, including Panton–Valentine leukocidin (PVL); and phenol-soluble modulins (PSMs) [6,7]. The main function of the toxins may be to convert local host tissues into nutrients needed for bacterial growth, and/or these toxins have critical effects on cells of the immune system to modulate and disturb the host’s immune response to *S. aureus.* SEs, TSST-1, PVL, and exfoliative toxin are implicated in heightened virulence among certain *S. aureus* strains. To date, numerous studies, including some from our research group, have demonstrated that exotoxins produced by *S. aureus* directly act on macrophages, mast cells, neutrophils, lymphocytes, and erythrocytes in both animals and humans. Epidemiological studies have found that specific MRSA lineages isolated from infectious diseases usually carry specific virulence genes. For example, ST9 and ST398 strains in MRSA often carry superantigen toxin genes, such as *sec* and *tst-1* exotoxin genes [8]. These findings underscore the critical role of exotoxins as essential virulence factors closely tied to the establishment and persistence of *S. aureus* infections. Previous articles have reviewed the function and pathogenicity of superantigen toxins as a paradox of the immune response and their ability to interact with the immune system as well as the capacity to be used as immunotherapeutic agents [1,9]. In this review, we focus on the current understanding of and emerging insights into the molecular characteristics, biological activities, and pathogenicity of *S. aureus*-produced exotoxins, including superantigen toxins; hemolysins and leukotoxins, which are membrane-damaging toxins; and exfoliative toxins.

## 2. Superantigen Toxins

SAg toxins are a large family of structurally related toxins, including SEs, SEls, and TSST-1. These toxins are the most potent T cell superantigens due to their ability to polyclonally activate T-cells at picomolar concentrations without the normal antigen presentation process. Whereas, staphylococcal protein A (SpA), produced by *S. aureus*, is also a superantigen that can interact with the antibody Fc and Fab regions of VH3+ immunoglobulins (Igs) of B cells.

### 2.1. Staphylococcal Enterotoxins and Staphylococcal Enterotoxin-like Toxins

SEs and SEls are proteins composed of approximately 168 to 261 amino acids, with a molecular size ranging from 19 to 30 kD. These toxins share common molecular structures, sequence homology, function, and phylogenetic relationships. Since the 1990s, more and more SEs have been revealed, and after 2001, many new types of SEs and SEls were discovered, one after another, by our research group [10,11,12,13] and other researchers [14,15]. To date, a total of 29 different SEs and SEls have been reported and described (Table 1), including SEA to SEE, SEG to SET, staphylococcal enterotoxin-like toxin U (SElU) to SElZ, SE01, SE02, SE26, and SE27. SEs and SEls have become a superfamily. To characterize and standardize family members, the International Nomenclature Committee for Staphylococcal Superantigens (INCSS) introduced standardized nomenclature for newly discovered toxins [16]. The naming convention established by INCSS underscores the importance of food poisoning, specifically emetic activity. For a toxin to be designated as a SE, it must demonstrate emetic activity through oral administration in a primate model. In cases where a toxin exhibits no emetic potential in primate vomiting experiments or when such experiments are yet to be conducted, the toxin should be labeled as a “SEl” toxin, even if the superantigen is structurally closely related to SE. Omoe et al. [13] conducted an assessment of the emetic potentials of several recently discovered SEls, including SElK, SElL, SElM, SElN, SElO, SElP, and SElQ, utilizing a monkey-feeding assay. The findings revealed that all tested SEls induced emetic responses in monkeys at a dose of 100 μg/kg. Subsequently, these newly identified SEls were re-designated as SEK, SEL, SEM, SEN, SEO, SEP, and SEQ, adhering to the naming convention of INCSS [13,17,18]. In a more recent study, Ono et al. reported the discovery of a novel staphylococcal emetic toxin, SElY, which exhibited robust emetic activity in a small emetic animal model, the house musk shrew [11].

#### 2.1.1. Genes of SEs and SEls

**Figure 1:**
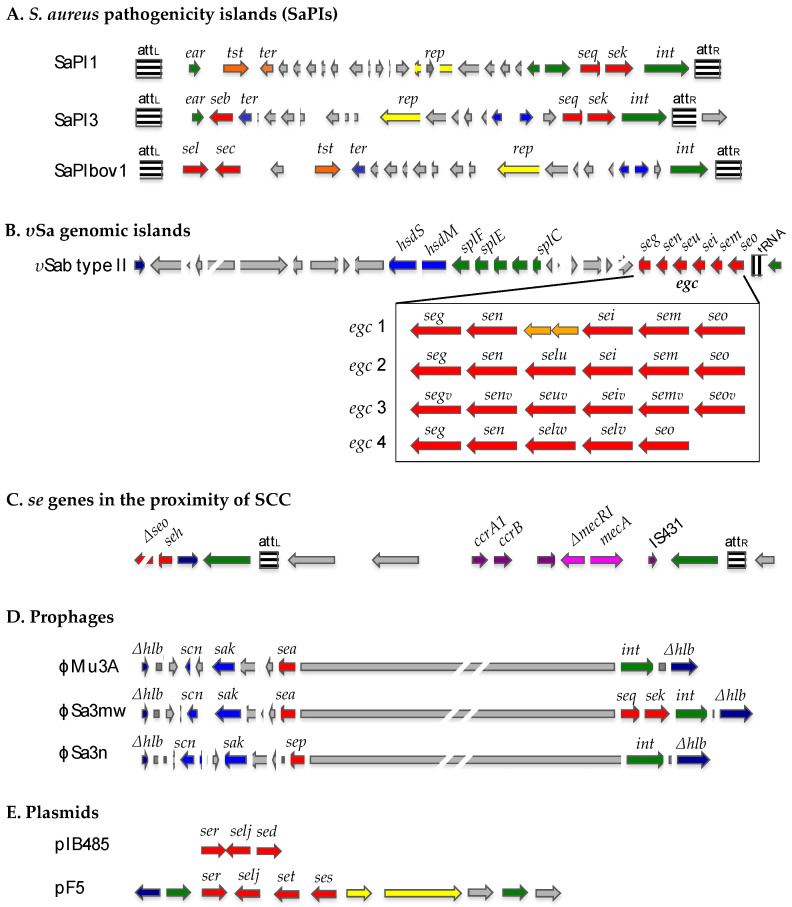
SE and SEl genes carried by SaPIs, υSa genomic islands, prophages, and plasmids based on sequencing data and modified from Novick and Subedi [23], Thomas et al. [14], Collery et al. [26], and Hu et al. [1]. different color of arrows means different genetic elements.

The genes of SEs and SEls are encoded by diverse accessory genetic elements, many of which are harbored in numerous mobile elements, including *S. aureus* pathogenicity islands (SaPIs), genomic islands (υSa), prophages, and plasmids (Figure 1) [6,19,20,21]. SaPIs other than SaPIbov2 (27 kb) and highly degenerate SaPI (3.14 kb) are present in some sequenced genomes [21,22]. Some SaPIs carry genes encoding one or more SEs. For instance, *sek* and *seq* are co-located with *tst* in SaPI1; *sel* and *sec* are found in SaPIbov1; and *seb*, *seq* and *sek* have been reported in SaPI3 [19,23]. Strains carrying different SaPIs exhibit significantly different production characteristics of superantigen toxins [19,21]. Genomic islands, υSaα and υSaβ, contain clusters of genes encoding virulence factors. υSaβ carries the enterotoxin gene cluster (*egc*), which includes a variable number of *se* or *sel* genes forming an operon. The enterotoxin gene cluster 1 (*egc*1) consists of five *se* genes (*seg*, *sei*, *sem*, *sen and seo*) [1,24,25]. The *egc*2 contains an additional *sel* gene (*selu*) [23]. Allelic variants of each of the *egc*2 genes compose the *egc*3 cluster [26,27], and two new *sel* genes (*selv*, *selw*) are present in *egc*4 [14]. SEs genes can also be carried by prophages [28,29,30,31]. Three *se/sel* genes (*sea, sek* and *seq*) are present together in ϕSa3ms and ϕSa3mw, while a single *se/sel* gene (*sea* or *sep*) is carried by ϕMu3A, ϕSa3a, and other prophages [30]. Two types of plasmids, pIB485 and pF5, carrying *se/sel* genes have been characterized [5,9,10,32]. pIB485 is a 27.6 kb plasmid, in which first *sed* and *selj* and latter *ser* were identified.

#### 2.1.2. Molecular Structures of SEs and SEls

**Figure 2:**
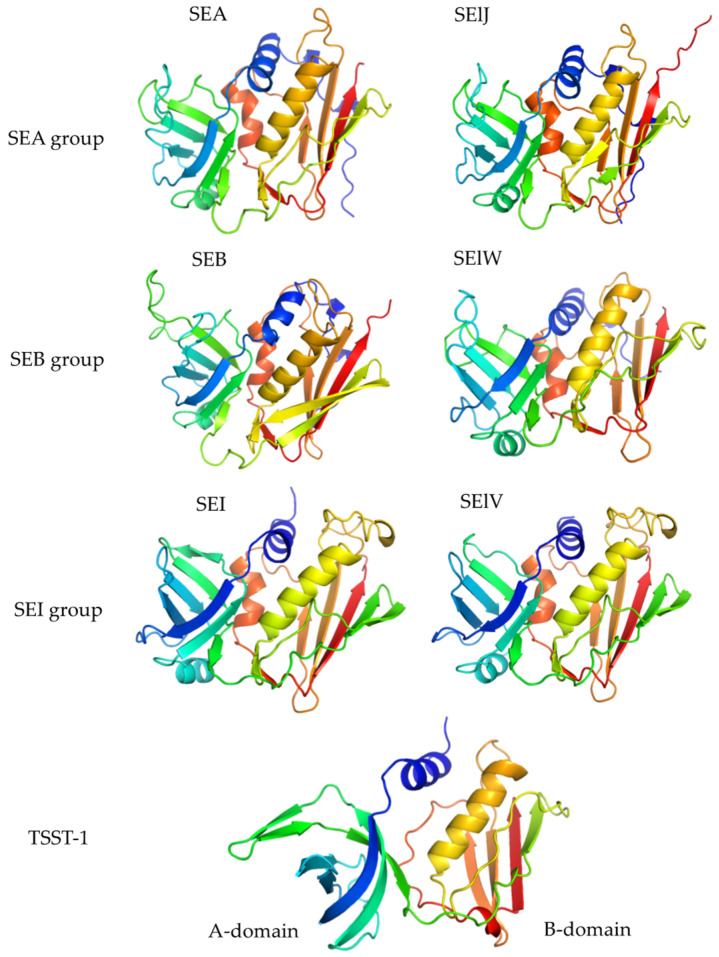
Molecular structures of SEs and SEls. SEs and SEls are single-chain proteins with molecular weights ranging from 19 to 30 kDa. The three-dimensional structures of SEs and SEls show very similar conformations wherein the canonical structure consists of one A domain, one B domain, and one α-helix that spans the center of the structure and connects the A and B domains.

SEs and SEls are single-chain proteins with two domains (A and B) and molecular weights ranging from 19 to 30 kDa (Figure 2). According to the homology of nucleotide and amino acid sequences, they can be classified into several groups. The SEA group, including SEA, SED, SEE, SEH, SElJ, SEN, and SEP, contains a cystine loop with nine amino acids [1,12,33]. These toxins possess a low-affinity α-chain major histocompatibility complex (MHC) II binding site and a high-affinity site known as the Zn^2+^-dependent β-chain MHC II binding site [34,35]. The presence of the Zn^2+^-dependent high-affinity site on the toxins makes them 10- to 100-fold more active overall in inducing cytokine production in T cells and antigen-presenting cells (APCs) than other superantigen toxins. The SEB group, which includes SEB, SEC, SEG, SElU, and SElW (SElU2), comprises a core superantigen structure plus a cystine loop with a variable 10- to 19-amino-acid sequence separating the cysteine residues [36,37,38,39]. These toxins contain one α-chain MHC II binding site, and this interaction does not depend on the interaction with the antigenic peptide within the MHC II peptide-binding groove [40,41]. The SEI group, including SEI, SEK, SEL, SEM, SEQ, SET, and SElV, contains both low- and high-affinity MHC II binding sites, but lack the cystine loop [42,43]. The three-dimensional structures of both SEs and SEls exhibit highly similar conformations. The canonical structure consists of one A domain and one B domain coupled with an α-helix spanning the center of the structure and connecting the A and B domains [44,45,46]. The interfaces between the A and B domains are characterized by a series of α-helices, creating an extended groove on the backside of the molecule and a shallow cavity at the top.

#### 2.1.3. Resistance of SEs and SEls to Heat and Enzymes

SEs and SEls are remarkably resistant to thermal and enzymatic degradation. The effectiveness of these toxins can only be diminished through extended boiling or autoclaving. They exhibit high stability against most proteolytic enzymes, ensuring their persistence in activity within the digestive tract post-ingestion. Various studies have assessed the comparative integrity and toxicity of SEA and TSST-1 following exposure to heat, pepsin, and trypsin, considering factors, such as food preparation conditions and their location in the stomach and intestinal lumen. SEA maintained significant superantigenic and emetic activity, even though it underwent degradation into smaller fragments after exposure to heat or pepsin. This implies that cooked food contaminated with these toxins could still lead to food poisoning [43,47,48].

#### 2.1.4. Superantigenic Activity of SEs and SEls

**Figure 3:**
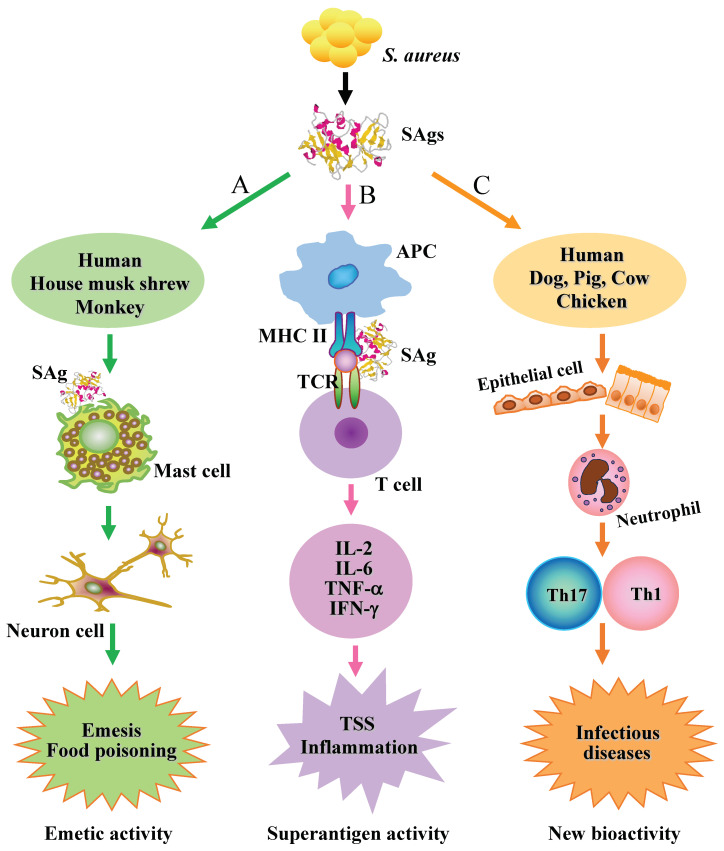
Biological activities of SEs and SEls. A. Emetic activity of SEs. Submucosal mast cells in the gastrointestinal tract are one of the target cells of SEs, and the serotonin released from mast cells and/or neuron cells plays an important role in SE-induced emesis and food poisoning. B. Superantigenic activity of SEs and SEls. Superantigens (SAgs), unlike conventional antigens, bypass normal processing by APCs, induce a large proportion of T-cells to proliferate, and subsequently stimulate a massive cytokine release that mediates the toxic effects of the toxins. C. Ability to cause infection diseases in different species using various cell pathways.

SEs and SEls, in contrast to typical antigens, circumvent regular processing by APCs and prompt a substantial proportion (5–30%) of T-cells to undergo proliferation, leading to extensive release of cytokines (Figure 3). The heightened release of cytokines, including tumor necrosis factor α (TNF-α), interleukin 1 (IL-1), IL-2, and gamma interferon (IFN-γ), is responsible for mediating the toxic effects induced by these toxins [34,49,50,51]. Each SE or SEl exhibits specificity towards a unique repertoire of Vβ-bearing T-cells, essentially forming a distinctive biological “fingerprint” [52,53,54]. SEs employ diverse modes of interaction with MHC class II molecules, as revealed by the crystal structure of SE in complex with MHC class II. SEA, for instance, possesses two MHC class II binding sites. The major interaction region is the zinc-dependent site in domain A, with crucial residues (H187, H225, and D227) for binding to MHC class II identified using mutagenesis experiments [1,52,55,56]. The second (minor) binding site on SEA is F47 located in domain B, which is not zinc dependent. The collaboration between these two binding sites potentially contributes to the high affinity of SEA for MHC class II [57,58]. However, no analogous site is predicted based on the structures of SEB and SEC. The binding of SEB to the MHC-peptide complex was examined using soluble HLA-DR1 loaded with hemagglutinin peptide HA 306–318. SEB exhibited a significantly higher affinity for the MHC class II molecule, potentially accounting for the observed differences in activity [59,60].

#### 2.1.5. Emetic Activity of SEs

SEs are the leading causes of foodborne bacterial intoxications worldwide [18,48,61,62,63]. SEA is most common enterotoxin recovered from food poisoning outbreaks in many countries [64,65,66]. The symptoms of food poisoning include vomiting, abdominal cramps, nausea, and occasionally diarrhea after a short incubation period [67,68]. Although the clinical manifestations are well-known, the mechanisms of SEs-induced emesis are only partially understood. Monkeys have been considered to be the primary animal model, but their use in investigating SEs is severely restricted due to the high cost, limited availability of the animals, and ethical considerations. Other animals, such as mice, rats, rabbits, and cats, are less susceptible to SEs or their responses to SEs are not specific [10,27,69,70]. Hu et al. investigated the emetic response of house musk shrews to both classic and several newly discovered SEls and showed that all tested SEs and SEls caused vomiting responses in the animals, albeit with varying emetic activities [9,43,68,71]. Ono et al. investigated the behavior of SEA in the gastrointestinal tract in vivo and suggested that the submucosal mast cells in the gastrointestinal tract are one of the cells targeted by SEA and that serotonin (5-HT) released from submucosal mast cells plays an important role in SEA-induced emesis [17,72,73] (Figure 3). The emesis in the house musk shrew is inhibited by the serotonin (5-HT) synthesis inhibitor and the 5-HT3 receptor antagonist, highlighting the important role of 5-HT in SEA-induced emesis [55]. SEA-induced emesis is blocked by surgical vagotomy in house musk shrews and primates, suggesting that 5-HT released from submucosal mast cells may bind to the 5-HT3 receptor expressed on enteric nerves in the gastrointestinal tract and thereby induce the depolarization of these nerves [55].

### 2.2. Toxic Shock Syndrome Toxin-1 (TSST-1)

Todd et al. originally reported a toxin that induces toxic shock syndrome (TSS) caused by *S. aureus* infection [74], which was initially named SEF. However, SEF lacks emetic activity, so it was renamed TSST-1. TSST-1 is a 22 kDa extracellular protein toxin secreted by *S. aureus*. Its physical and chemical properties are very similar to those of enterotoxins [47,75,76]. TSST-1 has unique primary amino acid sequences and contains only a low-affinity MHC II binding site in its O/B folds that interacts with the α-chains of MHC II molecules [76,77,78]. Toxin-activated T-cells/APCs leads to the release of various cytokines; enhances endotoxic shock; and causes T- and B-cell immunosuppression, fever, rash, vascular disorders, toxic shock syndrome, multiple organ failure and decrease blood pressure in both humans and animals.

### 2.3. Staphylococcal Protein A (SpA)

SpA produced by *S. aureus* is a B cell superantigen capable of interacting with both the antibody Fc and Fab regions of VH3+ immunoglobulins (Igs). The gene *spa* is encoded in the core genome of the majority of clinical isolates [79], and SpA proteins can be released from the bacterial cell wall by the hydrolase LytM in the cell wall [80]. The SpA molecule contains 4 to 5 highly conserved Ig-binding domains and X hypervariable regions composed of sub-regions Xr and Xc [81,82]. *S. aureus* isolates are classified based on the highly variable and repetitive octapeptides in Xr of SpA [79]. SpA has two distinct Ig-binding sites. One site is for IgG Fc domains, while a separate site binds an evolutionarily conserved surface on Fab encoded by VH3 clan-related genes. The Ig-binding domain on the SpA molecule confers the ability of SpA to bind to the Fcγ portion of the Ig and prevents opsonization of host’s cells [83]. The Ig-binding domains also mediate the binding of SpA to B cells by cross-linking VH3-expressing B cell receptors (BCRs) and thereby activating B cells. SpA exerts its mitogenic activity by binding to the variable regions of the heavy chain, rather than the complementarity-determining regions (CDRs) required for common antigens, thereby bypassing the antigen specificity required for B cell activation [84,85]. SpA may also represent a paradigm relevant to other microbial toxins with unconventional V region-targeted activity in *S. aureus* and other microbial commensal/opportunistic pathogens [86]. Such toxins with superantigen properties may be highly effective at subverting host defenses. SpA is required for persistence of *S. aureus* in the nasopharynx. Compared to animals colonized with wild-type *S. aureus*, mice colonized with the Δ*spa* variant mount increased IgG responses against staphylococcal colonization determinants. Immunization of mice with a nontoxigenic SpA variant, which cannot cross-link B cell receptors and divert antibody responses, elicits protein A-neutralizing antibodies that promote IgG responses against colonizing *S. aureus* and diminish pathogen persistence [87].

## 3. Membrane-Damaging Toxins

### 3.1. α-Hemolysin (α-Toxin or Hla)

**Figure 4:**
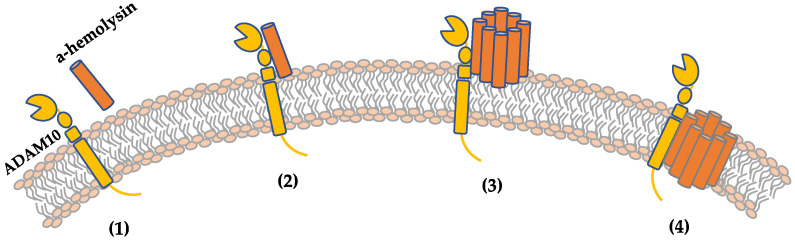
The hemolytic mechanism of α-hemolysin. (**1**) α-Hemolysin is a water-soluble monomer. (**2**) α-Hemolysin binds to the transmembrane protein ADAM10 that is a hemolysin receptor. (**3**) The toxin then oligomerizes at the plasma membrane to form heptamers and form pre-pores and (**4**) finally forms transmembrane channels.

α-Hemolysin, also termed hemolysin-α, α-toxin, or Hla, is a small β-pore-forming toxin (PFT) and is considered an important virulence factor of *S. aureus* in human and animal diseases. Encoded by a single genetic locus, *hla*, α-hemolysin is secreted as a 292-residue, 33.2 kDa water-soluble monomer. It is not heat-resistant and can be destroyed at 65 °C for 30 min. The regulation of α-hemolysin expression is mainly controlled by the quorum-sensing accessory gene regulator (*agr*) system, which is activated from the late logarithmic growth phase to the stationary phase of growth, thereby inducing the transcription and translation of *hla* [88,89]. In response to environmental conditions (such as the *SaeR/S* two-component system) or other systems (such as the *Sar* family), the *agr* system can also be adjusted to fine-tune *hla* expression [90]. After binding to its target cells, α-toxin oligomerizes to form a pre-porous structure and then attacks the cell membrane by extruding the β-barrel through the lipid bilayer, forming a hydrophilic transmembrane channel (Figure 4). α-Hemolysin assembles into a heptameric pore composed of three major domains: an extracellular-facing cap domain, a marginal domain that interacts with the extracellular leaflets of the host cell membrane, and a stem domain that forms a β-barrel transmembrane channel. The fully assembled toxin is 100 Å wide and 100 Å high, with a minimum pore diameter of 14 Å [91].

The hemolytic effect of α-hemolysin is cell-type specific because it selectively lyses rabbit red blood cells (RBCs), but does not lyse human RBCs [92]. However, α-hemolysin has been shown to lyse human platelets, endothelial cells, epithelial cells, and leukocytes [93]. *S. aureus* PFT receptors provide a powerful explanation for their cellular tropism, establishing the foundation for further studying the physiological effects of the toxins. Earlier studies showed that α-hemolysin bind and cleave protein-free membranes, suggesting that the α-hemolysin receptor is the lipid moiety and that binding is specific because the pore formation in liposomes depends on their lipid composition [94]. Liposomes composed of phosphatidylcholine (PC) or a combination of sphingomyelin and cholesterol are easily cleaved, while those with phosphatidylethanolamine (PE), phosphatidylserine (PS), phosphatidylglycerol (PG), or phosphatidylinositol (PI) composition are not [95,96]. Among them, PC appears to be a receptor for α-hemolysin because exogenous PC competes for t toxin binding, preventing hemolysis [96]. Several studies have shown that liposomes formed from lipids extracted from human or rabbit red blood cells have similar sensitivity to α-toxin, and treatment with amylase reduces the sensitivity of rabbit RBCs to the toxin, suggesting that the main receptor of α-hemolysin is a protein, but not a lipid.

Wilke and Wardenburg [97] used a biochemical method to identify cell surface metalloproteinases, a disintegrin, and metalloproteinase 10 (ADAM10) as the specific protein receptors for α-hemolysin. Loss of ADAM10 expression reduces α-hemolysin binding and cytotoxicity, while overexpression of ADAM10 increases binding and toxicity [98,99,100]. ADAM10 is expressed on cell types known to be susceptible to α-hemolysin poisoning, such as epithelial cells [101], keratinocytes [102], endothelial cells [103], and platelets [104]. Surface expression levels of ADAM10 on various cell lines correlate with Hla binding levels [97]. ADAM10 is a cell surface dehydrogenase involved in development and homeostasis processes [102,105]. Factors that regulate the surface expression of ADAM10 also regulate α-hemolysin sensitivity [99]. ADAM10 not only promotes pore formation, but its enzyme activity is also up-regulated by α-toxin pores [8].

α-Hemolysin is a β-barrel toxin secreted as a water-soluble monomer by 95% of clinical *S. aureus* strains [106,107]. This toxin has been shown to affect multiple human cell types, such as epithelial cells, endothelial cells, T cells, monocytes, and macrophages [108,109,110,111,112]. For many years, pore formation and cytolysis were considered to be the most prominent results of α-toxin action. However, recent studies have revealed its importance in altering cellular signaling pathways, including cell proliferation, inflammatory response, cytokine secretion, and intercellular interactions [93,108,113]. As the toxin approaches the cell, the pores formed allow ATP and K ions to be released quickly, while limiting the movement of macromolecules on the cell membrane [114,115]. The formation of pores leads to an influx of extracellular calcium into the cells, stimulating the hydrolysis of membrane phospholipids and the metabolism of arachidonic acid to leukotrienes, prostaglandins, and thromboxane A2 [116,117]. The toxin then also activates protein kinase K and induces NF-κB nuclear translocation [116,117]. All these events and the production of IL-1β, IL-6, and IL-8 act as pro-inflammatory stimuli. After the α-toxin bond disrupts the barrier function of the epithelial tissue, the enzyme that degrades E-cadherin is activated, which allows *S. aureus* to invade [118,119]. This toxin has been shown to play an important role in the pathogenesis of *S. aureus*, and the expression of α-toxin may be necessary for the pathogenesis of invasive diseases in healthy individuals [120,121].

### 3.2. β-Hemolysin (β-Toxin or Sphingomyelinase C)

The gene *hlb* encoding β-hemolysin is a part of the core genome of *S. aureus*. β-Hemolysin is a Mg^2+^-dependent neutral sphingomyelinase (SMase) that specifically cleaves sphingomyelin to produce ceramide and phosphocholine [122]. β-Hemolysin is a single-domain protein consisting of four layers: two β-sheet layers in the center and two alpha-helix and β-chains in the outer layer [123]. The crystal structure of β-hemolysin reveals structural homology with members of the DNase I superfamily [123]. This structural similarity suggests a secondary function of β-hemolysin. In addition, β-hemolysin enhances biofilm formation by catalyzing the formation of a nucleoprotein matrix in biofilms, making it a biofilm ligase [124].

In the presence of α-toxin-neutralizing serum, sheep erythrocytes are lysed, but not rabbit erythrocytes, and enhanced hemolysis is caused by shifting the temperature from 37 °C to a lower temperature. This unique phenomenon is due to membrane aggregation of ceramide hydrolysis products at 37 °C. When the temperature is lowered (i.e., 4 °C), phase separation occurs, resulting in the accumulation of ceramides in the pool, disruption of the lipid bilayer, and altering the erythrocyte membrane structure [125]. Therefore, β-hemolysin is also called a hot-cold hemolysin.

β-Hemolysin exhibits species-related hemolytic activity. Red blood cells from sheep, cattle, and goat are highly sensitive to the toxins. Rabbits and humans are moderately sensitive, and mouse and dog red blood cells are resistant. The difference in sensitivity among different red blood cells may be due to variations in sphingomyelin content. The activity of β-hemolysin SMase also causes the lysis of human keratinocytes, monocytes, T cells, and bovine epithelial cells [123,126,127]. β-Hemolysin stimulates the production of proinflammatory cytokines in human monocytes (158) but inhibits the production of IL-8 and the expression of cell adhesion molecules in human endothelial cells; thus, the toxin prevents the migration of leukocytes across endothelial cells [128].

β-Hemolysin is produced in large quantities in strains isolated from bovine mastitis [89] and chronic skin infections [90]. Several studies have demonstrated the importance of this toxin for the pathogenicity of *S. aureus*. Infections with β-hemolysin-producing *S. aureus* can cause larger lesions in organs [129]. The presence of β-hemolysin enhances the colonization of *S. aureus* on the skin during keratitis in mice [127] and causes damage to scleral epithelial cells [90]. Intranasal injection of β-hemolysin induced shedding of lung epithelial cells, syndecan-1 production, and infiltration of neutrophils into the lungs of mice [130]. In pneumonia and mouse ear skin infection models, the toxicity of mutant *S. aureus* strains lacking HLB is reduced [127,130].

### 3.3. Leukotoxin

**Figure 5:**
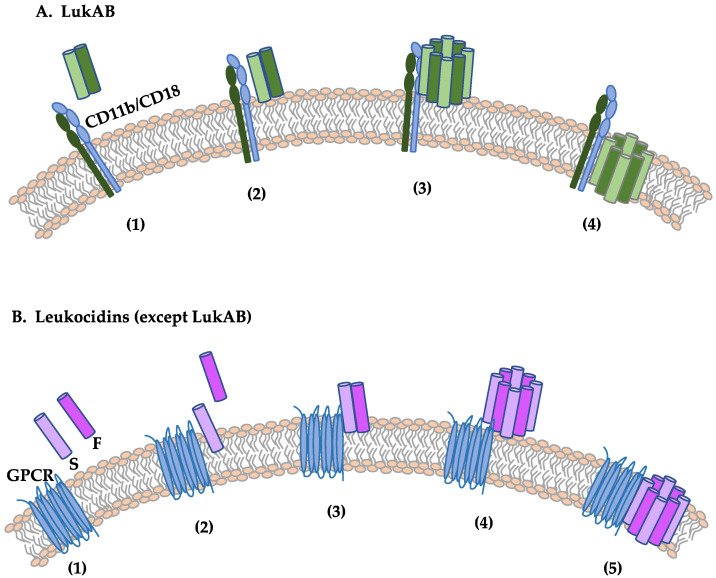
Mechanism of action of leukotoxin. (**A**) The bicomponent leukotoxin, LukAB. (**1**) LukAB is secreted as dimers. (**2**) LukAB recognizes the target cell by binding to cell surface receptors, namely, the integrin, CD11b. (**3**) Upon receptor binding, they dimerize with additional leukocidin dimers to form an octameric pre-pore. (**4**) The prestem domains of the pre-pore extend to form a β-barrel pore, which eventually destroys the target cell membrane. (**B**) Bicomponent leukocidins (except LukAB). (**1**) The leukotoxins are secreted as monomers. (**2**) The S subunit recognizes the target cell by binding to cell surface receptors that are typically GPCRs. (**3**) The S subunit dimerizes with the F subunit. (**4**) These dimers oligomerize at the plasma membrane and a pre-pore appears. (**5**) Finally, transmembrane channels are formed, thus disrupting the target cell membrane.

Leukotoxins lyse cells of the leukocytic lineage and are also known to kill neutrophils, of which γ-hemolysin and LukED also exhibit lytic activity against red blood cells [131,132,133]. Leukotoxins are bicomponent pore-forming toxins (PFTs) composed of two different protein components that assemble together to form β-barrel pores [134,135]. They share structural homology with α-toxin and have a similar pore formation mechanism (Figure 5). Bicomponent PFTs require two subunits: the fast-eluting subunit, F subunit, and the slow-eluting subunit, S subunit [136]. The S subunit recognizes and binds to a surface receptor on target cells, recruiting the F subunit for dimerization [106,137]. Before the insertion of the β-barrel transmembrane channel into the target cell membrane, oligomerization occurs to form the pre-pore, resulting in cell lysis [134,138]. Similar to the α-toxin heptamer, the bicomponent PFT octamer also resembles a mushroom, consisting of the cap, rim, and stem domains.

Leukotoxins mainly target leukocytes, earning them the name leukocidins (Luk). In additional to mediating cytolysis, many leukocidins also have sublytic effects, causing extracellular Ca^2+^ influx in host cells and the production of proinflammatory cytokines [139,140,141,142]. Several leukocidins, PVL, HlgAB, and LukAB, stimulate K^+^ efflux, the production of the NLRP3-inflammasome, and activation of caspase-1, resulting in a form of inflammatory cell death known as pyroptosis. At present, four leukotoxins have been identified from *S. aureus* strains associated with human infections: (1) γ-hemolysins AB and CB (HlgAB, HlgCB); (2) LukSF-PV (Panton–Valentine leukocidin, PVL); (3) leukotoxin ED (LukE, LukD); and (4) leukotoxin AB/GH (LukAB/LukGH) [131]. Two of the leukotoxins, LukMF’ and LukPQ, are associated with animal infections [143,144].

#### 3.3.1. γ-Hemolysins (HlgAB, HlgCB)

The γ-hemolysin locus is part of the core genome of *S. aureus* and is present in approximately 99% of the sequenced *S. aureus* genome [145,146]. It encodes three genes: *hlg*ACB, comprising two S subunits, HlgA and HlgC, and one F subunit, HlgB. *hlg*CB forms one transcription unit, and hlgA forms another transcription unit, each with an independent promoter [147]. Within the γ-hemolysin group, HlgAB, HlgCB and HlgACB are three proteins with different combinations of subunits. HlgAB and HlgCB share the same F subunit (HlgB), but S subunit composition is different (HlgA or HlgC) [131]. HlgA and HlgB combine to form the classic γ-hemolysin (HlgAB). HlgAB is particularly effective in lysing human red blood cells and shows cytolytic activity on human and rabbit leukocytes [147,148,149]. Although HlgCB is also known as γ-hemolysin, it is mainly leukotoxic and has limited activity towards red blood cells. Early studies referred to it simply as leukocidin (LukSF) or leukocidin R (LukR) [149,150].

HlgAB exhibits tropism towards human red blood cells, neutrophils, and macrophages, leading to their lysis. Human CXCR1, CXCR2, CCR2, and CXCR4 (at very high concentrations) were identified as receptors for HlgA [149,151]. HlgAB can also target and lyse murine monocytes and macrophages through CCR2, but does not lyse murine neutrophils. Here are two explanations: (1) mouse neutrophils mainly express CXCR2, whereas human neutrophils mainly express CXCR1; (2) murine CXCR2 does not bind the toxins [151]. Thus, the host tropism of the toxin depends not only on structural compatibility of the receptors, but also on differences in gene expression in the cells. Duffy antigen receptor for chemokines (DARC) is also a receptor for HlgAB [149]. HlgA and LukE have different binding sites on DARC. Many polymorphisms of the DARC gene, coding the region of the receptor responsible for antigenic differences, exist in humans. Antigen differences have no effect on HlgA or LukE binding, but DARC expression levels are related to hemolysis [149]. HlgCB, on the other hand, is a human-specific toxin that targets cells expressing the receptors C5aR1 and C5aR2 [151].

The role of HlgACB in the virulence of *S. aureus* remains unclear. Some studies suggest that HlgAB is necessary for the survival and proliferation of *S. aureus* during a blood infection, which is most likely caused by macrophage escape and nutrients (Fe^2+^) released by red blood cells [149,151]. Approximately 89–100% of the strains associated with human or bovine colonization contain the hlgACB locus, but evidence associated with any particular type of infection has not been reported [152,153]. γ-Hemolysin causes acute tissue damage and inflammation in different animal models and causes *S. aureus* disease. Post-orbital administration of micrograms of HlgAB is fatal to mice. Intravitreal injection of γ-hemolysin into rabbits is highly toxic, causing eye and tissue damage [154]. Tissue damage may be the result of a combined effect of toxin-mediated cell lysis and apoptosis due to excessive concentration of toxins [155]. The contribution of HlgAB to the disease has been further demonstrated in several infection models with the strains that do not produce HlgAB [151,156,157].

#### 3.3.2. Panton–Valentine Leukocidin (LukF-PV and LukS-PV)

The Panton–Valentine Leukocidin (PVL) genes (lukS-PV and lukF-PV) are encoded within the genomes of at least six different prophages (e.g., ϕPVL, ϕPVL108, ϕSa2mw, ϕSa2USA300, ϕSLT) expressing the Sa2 integrase [158]. Initially purified from the culture supernatants of *S. aureus* V8, PVL-producing isolates express class S (slow-eluted) and class F (fast-eluted) proteins specific for PVL (LukS-PV and LukF-PV) [158,159]. PVL exhibits species specificity, selectively killing rabbit and human leukocytes. This specificity stems from its targeting of human and rabbit G-protein-coupled receptors (GPCRs), C5aR1 and C5aR2, while not affecting their murine counterparts [160,161] (Figure 5). Consequently, rabbit models and human ex vivo models offer valuable insights into the intricacies of PVL-mediated pathology, with regular mice proving unsuitable for studying this toxin.

PVL is a cytotoxin that impacts leukocytes, contributing to tissue necrosis, and is associated with furuncles, cutaneous abscesses, and severe necrotic skin infections [162,163]. It plays a crucial role in causing invasive diseases, such as osteomyelitis and pneumonia, in rabbits. Removal of *pvl* has been shown to reduce inflammation, tissue damage, and bacterial burden and enhance host survival [164,165]. Sublytic levels of PVL can augment phagocytosis and bacterial killing by primary human neutrophils [166]. However, the role of PVL in skin and soft tissue infections remains unclear, with some studies suggesting its pathogenic impact could be contingent on the site of infection [167,168].

The contribution of PVL to the virulence of *S. aureus* has not yet been confirmed [98]. Molecular epidemiological studies have found that PVL accounts for a small percentage (5%) of clinical *S. aureus* strains, but is closely related to community-acquired MRSA (CA-MRSA) strains (85%), especially in the strains causing pneumonia, skin and soft tissue Infections [131]. Phylogenetic analysis of CA-MRSA strains reveals that multiple strains containing staphylococcal cassette chromosome mec (SCCmec) IV and PVL, rather than a single virulent clone, have arisen around the world, corroborating the possibility that the virulence associated with CA-MRSA could be horizontally transferred [169]. Clinical studies have shown that pneumonitis involving PVL-positive *S. aureus* progresses faster and is more lethal than PVL-negative pneumonia [170]. Autopsy revealed severe inflammation with ulcers and bleeding in the lungs of these patients, attributed to PVL-induced lysis of macrophages and neutrophils [170].

#### 3.3.3. LukAB/GH

LukAB, also known as LukGH, is the most recently discovered member of the bicomponent leukotoxin family [86]. It is also the most distantly related member of the bicomponent leukocidin family, with LukA exhibiting only 30% amino acid identity with other S subunits and LukB only demonstrating 40% identity to other F subunits [86,106]. LukAB/GH heterodimerize before binding to its cell-surface receptor, suggesting that dimerization occurs prior to secretion, as the co-expression of subunits appear to be more toxic than the mixture of subunits individually purified [171,172] (Figure 5). This is different for other leukotoxins that are secreted as monomers and oligomerize after binding to the cell surface.

The *luk*AB locus is part of the core genome of *S. aureus* and is found in 99% of *S. aureus* strains. A large number of LukAB is found in the secreted proteome during the late exponential growth phase of *S. aureus* [86,173]. The C-terminal region of LukA is critical for toxin activity, as deletions or mutations within this region render the toxin inactive [171]. LukAB has species specificity. It is most effective in human and primate cells, followed by rabbit, while its activity is ~1000-fold lower in mice [174]. LukAB mediates cytotoxicity by targeting the I-domain of the CD11b receptor present on leukocytes, including neutrophils, monocytes, macrophages, dendritic cells, and NK cells [171,175].

The role of LukAB during infection remains to be fully elucidated. This toxin appears to be encoded in most sequenced *S. aureus* strains [176]. Higher LukAB-neutralizing antibody titers were observed in the sera of patients with invasive *S. aureus* disease, indicating that LukAB is produced during infection [177,178]. In vitro and ex vivo studies have provided insights into the role of LukAB in diseases. Primary human neutrophils infected with ΔlukAB strains exhibit enhanced survival compared to wild-type, suggesting that the virulence of lukAB-deficient mutants is greatly reduced [86]. LukAB can promote survival of *S. aureus* in leukocytes, which may be related to the role of the toxins in escaping from phagocytes and neutrophils [86,179].

#### 3.3.4. LukED

LukED was originally reported more than two decades ago. The *luk*ED locus is located in the νSaβ gene cluster, and the two genes *luk*E and *luk*D in this locus are co-transcribed at the late exponential phase [180]. Epidemiological data indicate that *luk*ED is widespread, especially in prevalent CA-MRSA strains (~99%) [133,181]. The *luk*ED locus is also present in approximately 90% of *S. aureus* strains isolated from cows with subclinical to severe mastitis, suggesting that LukED may play a role in virulence or infectivity in different host species [182].

Earlier investigations have indicated that LukED exhibits cytolytic activity against both rabbit and human erythrocytes and leukocytes [133]. Further research has confirmed that LukED is capable of mediating the lysis of various human and murine bone marrow-derived cells, and, notably, it is the only toxin efficient in killing mouse phagocytes [183]. LukED displays a broad spectrum of leukocyte-killing activities, and its pathogenic effects are receptor dependent [184]. LukED can target G protein-coupled receptors on neutrophils, monocytes, macrophages, dendritic cells, NK cells, T-cells, and red blood cells [149,184]. Treatment of a range of human cell lines with purified LukED revealed cytotoxicity in T-cell lines expressing CCR5. Knockdown and overexpression studies confirmed that CCR5 is a receptor for LukED [185]. The S-component LukE, but not LukD, directly interacts with the receptor [185]. Furthermore, CXCR1 and CXCR2 were identified as two additional LukE receptors expressed in PMNs and monocytes [184].

LukED serves as a crucial virulence factor in *S. aureus* infection. Investigations have demonstrated that LukED induces dermonecrosis of rabbit skin [180]. Administering LukED through retroorbital injection leads to acute lethality in mice [186]. Moreover, the virulence of a Δ*lukED* strain was significantly attenuated, resulting in reduced inflammation, a lower bacterial burden, and improved host survival in murine infections [184,185].

#### 3.3.5. LukMF’

LukMF’ expression is restricted to *S. aureus* strains transmitted among non-human hosts, such as cattle, sheep, and goats (134). LukMF’ was isolated from bovine mastitis tissue samples, suggesting its common association with bovine mastitis, which is a major cause of milk loss and a global economic problem. These data indicated a role of LukMF’ in the progression of bovine mastitis [134,135]. The *luk*MF’ locus is encoded in the temperate prophage ΦSa1, which can spread horizontally across strains, much like PVL [144]. However, unlike other leukocidins, purified LukMF’ does not induce a strong proinflammatory response when incubated with primary bovine macrophages [187].

LukMF’ is well-adapted to the bovine host and shows cytolytic activity towards bovine neutrophils and macrophages [188]. Compared with other species, LukMF’ is particularly toxic to PMNs of bovine, indicating a possible host-specific adaptation [134]. LukM is able to bind surface receptors CCR1, CCR2, and CCR5 of the cells and infect bovine macrophages and neutrophils [188]. However, LukM binding is restricted to CCR1 and macrophages in humans. The tropism of LukM is primarily derived from the DR4 loop of the fringe domain; thus, it binds to bCCR1 and bCCR2 but does not affect the binding of bCCR5 [188].

#### 3.3.6. LukPQ

LukPQ is a novel equid-adapted leukocidin of *S. aureus*. LukPQ is encoded by a 45 kb prophage (ΦSaeq1), almost exclusively in strains isolated from horses. Like other bicomponent PFTs, LukPQ preferentially targets leucocytes and consists of an S and an F subunit. The S subunit recognizes the receptor on host cells, enabling high-affinity binding to the cell surface. Then, the toxins form a pore that penetrates the lipid bilayer of the cells.

LukPQ is an effective and specific killer of equine neutrophils. Equine neutrophils are most sensitive to the lysis of LukPQ followed by bovine neutrophils, whereas human neutrophils are relatively insensitive to the toxin [189] (Figure 3). Although the S subunit (LukP) is highly similar to that of LukED, the species specificities of LukPQ and LukED are different. Uniquely, the F subunit of LukQ is responsible for conferring species specificity of LukPQ, whereas, species specificity is conferred by the S subunit in other leukocidins [189]. Equine GPCRs, CXCRA and CXCR2, are the target receptors for LukPQ. At high concentrations, the toxin also targets equine CCR5. LukPQ binding can be inhibited by cytokines binding to these receptors, suggesting a shared binding site between LukP and the receptor ligands [189].

#### 3.3.7. ε-Toxin

ε-Toxin is a newly identified cytotoxin secreted by a surgical site isolate of *S. aureus* that lacks homology to known exotoxins [190]. The gene encoding ε-toxin, *cytE*, is conserved in the core genome of *S. aureus*. The toxin has a molecular weight of 15.7 kDa and an isoelectric point of 8.9. The cloned and purified protein has cytotoxic and proinflammatory properties. As shown in vitro and in vivo, ε-toxin lyses rabbit erythrocytes and human keratinocytes.

Lytic concentrations of ε-toxin in keratinocytes promote the secretion of the pro-inflammatory cytokine IL-8 [190]. In contrast, lower concentrations of ε-toxin slow the proliferation of keratinocytes. Subcutaneous administration of microgram amounts of ε-toxin can cause neutrophil recruitment to the injection site in rabbits and also causes rabbits to develop fever after intravenous administration of the toxin [190]. The potent biological effects on keratinocytes and rabbit skin suggest that the toxin may play an important role in impairing normal wound healing and preventing re-epithelialization.

#### 3.3.8. Phenol Soluble Modulins (PSMs)

Phenol soluble modulins (PSMs) are amphipathic peptides uniquely found in *Staphylococci*. Three peptides, PSMα, PSMβ, and PSMγ, were identified by hot phenol extraction from the culture filtrate [191,192]. PSMγ is the same as the previously described *S. epidermidis* δ-toxin. In *S. aureus*, PSMs are encoded by three loci of the core genome [192]. PSMα peptides (PSMα1-PSMα4) are encoded by the *psmα* operon; PSMβ (PSMβ1, PSMβ2) peptides are encoded by the *psmβ* operon; and PSMγ (δ-toxin) is encoded by *hld*, which is also part of the coding sequence for RNAIII [193]. PSMs are secreted with an N-terminal N-formylmethionine but without a signal peptide [192], indicating a dedicated mechanism of secretion, which was identified to be the four-component ABC transporter, phenol-soluble modulin transporter (Pmt).

**Figure 6:**
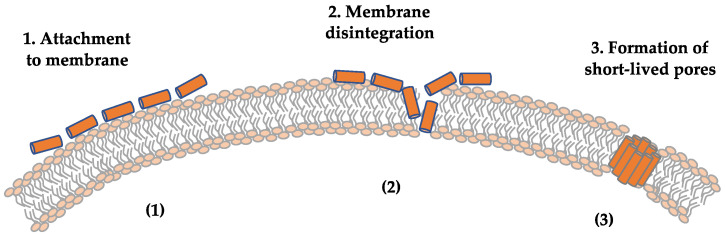
Pore-formation mechanism of phenol-soluble modulins (PSMs). (**1**) PSMs attach the cytoplasmic membrane in a non-specific way. (**2**) The attachment can lead to membrane disintegration. (**3**) PSMs aggregate in oligomers and form a short-lived pore.

PSMs are categorized into 2 types based on amino acid length [193]. PSMα and δ-toxin are α-type PSMs, which are typically 20–25 amino acids long, have a neutral or positive net charge, and form one α-helix [194]. In contrast, PSMβ2 belongs to the β-type PSMs. They are longer, typically 43–45 amino acids in length, having a negative net charge, and forming 3 α-helices that fold to a “v”-like shape [194]. PSM peptides form an α-helix amphipathic structure, which stretches over virtually the entire length of the peptide in the shorter α-type PSMs and are located in the carboxy-terminal region in the longer β-type PSMs. PSMs have different charge characteristics: PSMα are positively charged, PSMβ are negatively charged, and δ-toxin is neutral [193]. PSMs can attach to the cytoplasmic membrane in a non-specific manner and cause the membrane to disintegrate [195] (Figure 6). Phospholipid composition and membrane charge are important for cell susceptibility to PSMs [195]. PSMs tend to aggregate as oligomers, forming short-lived pores.

PSMs have multiple roles in the pathogenesis of *S. aureus*, including cell lysis, biofilm formation, and immune modulation. The ability to promote surface diffusion or form biofilms appears to be a major aspect of the pathogenesis [193]. The transcription product of the *psm-mec* gene, located in the mobile genetic element SCCmec of HA-MRSA, but not CA-MRSA, suppresses the expression of phenol-soluble modulin α (PSMα), a cytolytic toxin of *S. aureus* [196]. PSMs have a key impact on the capacity of virulent *S. aureus* to cause skin infections and bacteremia in animal infection models. α-Type PSM peptides have high potency in lysing eukaryotic cells in a receptor-independent manner by targeting cell membranes [192,197]. Phagocytosed *S. aureus* produces PSMs to lyse neutrophils and osteoblasts intracellularly, indicating a role in the intracellular escape of *S. aureus* [197,198].

PSMs have immune modulatory effects on host cells. At nanomolar concentrations, they stimulate leukocytes and initiate pro-inflammatory responses, including neutrophil chemoattraction, activation, and IL-8 release. The neutrophil-attracting properties of PSMs are important in local *S. aureus* infections and contribute to inflammation (Figure 4). In humans, the pattern recognition receptor formyl peptide receptor 2 (FPR2) can bind to PSM [199]. FPR2 is a member of the G-protein-coupled receptor family that specializes in recognizing pathogen-associated molecular patterns (PAMPs) produced by bacteria. FPR2 is predominately expressed on innate immune cell types, including neutrophils, monocytes, macrophages, and immature dendritic cells. FPR2 not only detects PAMPs, but also monitors the invader’s pathogenicity to properly regulate the immune response. After activation by PSMs, FPR2 induces a series of pro-inflammatory responses, including leukocyte activation, neutrophil chemotaxis, and cytokine production [200].

PSMs also have biofilm-structuring activities and influence biofilm development via their shared physico-chemical properties. Biofilm plays a significant role in staphylococcal infections by increasing adherence and colonization, antibiotic resistance, and virulence factor production. PSM expression can also lead to biofilm dispersal, i.e., the detachment of cells or cellular clusters from biofilms, which is a key mechanism leading to the systemic dissemination of biofilm infection. These characteristics contribute to the sustained infection effect of *S. aureus*, thereby escalating the challenges associated with the treatment of *S. aureus* infections [201].

## 4. Exfoliative Toxins

Exfoliative toxins (ETs), also known as epidermolytic toxins, are extremely specific serine proteases secreted by *S. aureus*. The principal ETs include ETA, ETB, ETC, ETD, and ETE according the antigenically distinct forms. The gene of each ET is encoded on a different mobile genetic element. Specifically, *eta* is encoded by the genome of a temperate phage [202], and *etb* is found on the plasmid pETB. In contrast, *etc* has not been described, although t ETC was purified from a *S. aureus* isolate associated with horse infection. Moreover, *etd* is encoded as part of a pathogenicity island [203], and *ete*, the most recently characterized *S. aureus* ET that was previously termed ETD-like, was discovered in *S. aureus* isolates from ewe mastitis [204].

ETs are glutamate-specific serine proteases of the chymotrypsin family. The catalytic triad (histidine, aspartate, serine) is conserved across all ETs [205]. ETA and ETB, with structural similarities and homology, require N-terminal α-helical extension for enzyme activity [206]. The crystal structures of both ETA and ETB represent the inactive forms of the enzymes, suggesting that the protease activity of ET may require a specific cellular target and occur under specific conditions [206]. ETs recognize and induce hydrolysis of desmosome cadherins in the superficial layers of the skin [205]. ETs can cleave keratinocytes junctions and cell–cell adhesion in the epidermis of the host, inducing skin peeling and blister formation [201,207]. ETA and ETB are the most implicated in human skin damage, while ETC was only isolated from a horse infection and exhibits no association with human disease [204]. ETD was only identified in a clinical sample of *S. aureus*.

Approximately 5% of *S. aureus* strains produce ETs [208]. The production of ETs in certain strains is related to localized epidermal infections and generalized diseases, such as staphylococcal scalded skin syndrome (SSSS) including Ritter’s disease, toxic epidermal necrosis, bullous impetigo, and certain erythema cases [204]. ETs interact with human and mouse desmoglein 1 (Dsg1), causing blistering of the superficial skin [207,209]. The effects of ETs can manifest widely across the body, predominantly in neonates, infants, and immunocompromised adult patients [204].

The syndromes of SSSS are characterized by the formation of blisters and superficial desquamation and skin exfoliation, but its early manifestations include fever, skin hypersensitivity, and erythema, followed by superficial fluid-filled blister formation and skin separation [204,209]. The lesions characteristic of SSSS are often sterile because the ETs can be distributed through the bloodstream from a distant site to cause symptoms [210].

## 5. Concluding Remarks and Future Perspective

Although there is currently a relatively comprehensive view of *S. aureus* exotoxins, further in-depth research remains imperative. An in-depth understanding of exotoxins and their pathogenic mechanisms helps us uncover new therapeutic targets and develop effective vaccines. The rise of multidrug-resistant *S. aureus* necessitates the development of alternative antimicrobial strategies. Exploring the potential of new compounds and treatment modalities is crucial in managing these infections. Further research into host–microbe interactions will shed light on genetic and immunological factors that influence susceptibility to *S. aureus* infections in both humans and animals.

In summary, the extensive arsenal of virulence factors is crucial for the survival and infection process of *S. aureus*, and the future of combating it relies on a multidisciplinary approach that integrates microbiology, immunology, and clinical research. The development of effective prevention and treatment strategies is not only crucial for human well-being but also for safeguarding the health of livestock and the sustainability of agriculture. It is imperative that researchers, healthcare professionals, and veterinarians continue to collaborate in addressing this pressing global health challenge.

## Figures and Tables

**Table 1:** Molecular characteristics and pathogenicity of staphylococcal superantigen toxins *.

Types	Molecular Weight (kDa)	Superantigen Activity	Genetic Elements	Emetic Activity ^(1)^		First Reported
				Monkey ^(2)^	Shrew ^(3)^	
SEA	27.1	+	Prophage	25	0.3	1963
SEB	28.3	+	Chromosome, SaPI3, Plasmid (pZA10)	100	10	1963
SEC1	27.5	+	SaPI	5	NE	1965
SEC2	27.6	+	SaPI	NE	1000	1965
SEC3	27.6	+	SaPI	<50	NE	1965
SED	26.9	+	Plasmid (pIB485)	NE	40	1967
SEE	26.4	+	Prophage (Hypothetical location)	NE	10	1971
SEG	27	+	*egc*1, *egc*2, *egc*3, *egc*4	160–320	200	1998
SEH	25.1	+	Transposon (MGEmw2/mssa476 *seh*/Δ*seo*)	30	1000	1994
SEI	24.9	+	*egc1*, *egc*2, *egc*3	300–600	1	1998
SElJ	28.6	+	Plasmid (pIB485, pF5)	NE	NE	1998
SEK	25.3	+	Prophages, SaPI1, SaPI3, SaPI5, SaPIbov1	100 (2/6)	1000	1998
SEL	24.7	+	SaPIn1, SaPIm1, SaPImw2, SaPIbov1	100 (1/6)	500	2001
SEM	24.8	+	*egc*1, *egc*2*,*	100 (1/7)	100	2001
SEN	26.1	+	*egc*1, *egc*2, *egc*3, *egc*4	100 (1/8)	1000	2001
SEO	26.8	+	*egc*1, *egc*2, *egc*3, *egc*4, Transposon	100 (2/6)	500	2001
SEP	26.7	+	Prophage (Sa3n)	100 (3/6)	50	2001
SEQ	25.2	+	SaPI1, SaPI3, SaPI5, Prophage	100 (2/6)	4	2002
SER	27	+	Plasmid (pIB485, pF5)	<100	<1000	2003
SES	26.2	+	Plasmid (pF5)	<100	20	2008
SET	22.6	+	Plasmid (pF5)	<100	1000	2008
SElU	27.2	+	*egc*2, *egc*3	NE	NE	2003
SElV	26.7	+	*egc*4	NE	NE	2006
SElW	27.3	+	*egc*4	NE	NE	2006
SElX	19.5	+	Chromosome	NE	NE	2011
SEY	23.3	+/−	Chromosome	250	500	2015
SElZ	27.4	+	Chromosome	NE	NE	2015
SE01	26.4	+	Plasmid	NE	NE	2017
SE02	27.2	+	Chromosome	250	NE	2020
SEl26	25.1	+	SaPI	NE	NE	2018
SEl27	27.1	+	SaPI	NE	NE	2018
TSST-1	22.1	+	SaPIs	-	-	1981

+ Positive reaction, − Negative reaction, ^(1)^ mg/animal, ^(2)^ Oral administration, ^(3)^ Intraperitoneal administration, NE: Not examined. * This table is modified from Hu, et al., 2021 [1].

## References

[1] Hu D.L., Li S., Fang R., Ono H.K. (2021). Update on molecular diversity and multipathogenicity of staphylococcal superantigen toxins. Anim. Dis..

[2] Bourély C., Cazeau G., Jarrige N., Leblond A., Madec J., Haenni M., Gay E. (2019). Antimicrobial resistance patterns of bacteria isolated from dogs with otitis. Epidemiol. Infect..

[3] Hu D.L., Omoe K., Sasaki S., Sashinami H., Sakuraba H., Yokomizo Y., Shinagawa K., Nakane A. (2003). Vaccination with nontoxic mutant toxic shock syndrome toxin 1 protects against *Staphylococcus aureus* infection. J. Infect. Dis..

[4] Hu D.L., Omoe K., Narita K., Cui J.-C., Shinagawa K., Nakane A. (2006). Intranasal vaccination with a double mutant of staphylococcal enterotoxin C provides protection against *Staphylococcus aureus* infection. Microbes Infect..

[5] Hu D.L., Nakane A. (2014). Mechanisms of staphylococcal enterotoxin-induced emesis. Eur. J. Pharmacol..

[6] Tam K., Torres V.J. (2019). *Staphylococcus aureus* secreted toxins and extracellular enzymes. Microbiol. Spectr..

[7] Oliveira D., Borges A., Simoes M. (2018). *Staphylococcus aureus* toxins and their molecular activity in infectious diseases. Toxins.

[8] Zhu Z., Wu S., Chen X., Tan W., Zhou G., Huang Q., Meng X., Hu D.L., Li S. (2023). Heterogeneity and transmission of food safety-related enterotoxigenic *Staphylococcus aureus* in pig abattoirs in Hubei, China. Microbiol. Spectr..

[9] Truant S.N., Redolfi D.M., Sarratea M.B., Malchiodi E.L., Fernández M.M. (2022). Superantigens, a paradox of the immune response. Toxins..

[10] Ono H.K., Omoe K., Imanishi K., Iwakabe Y., Hu D.L., Kato H., Saito N., Nakane A., Uchiyama T., Shinagawa K. (2008). Identification and characterization of two novel staphylococcal enterotoxins, types S and T. Infect. Immun..

[11] Ono H.K., Sato’o Y., Narita K., Naito I., Hirose S., Hisatsune J., Asano K., Hu D.L., Omoe K., Sugai M. (2015). Identification and characterization of a novel staphylococcal emetic toxin. Appl. Environ. Microbiol..

[12] Suzuki Y., Ono H.K., Shimojima Y., Kubota H., Kato R., Kakuda T., Hirose S., Hu D.L., Nakane A., Takai S. (2020). A novel staphylococcal enterotoxin SE02 involved in a staphylococcal food poisoning outbreak that occurred in Tokyo in 2004. Food Microbiol..

[13] Omoe K., Hu D.L., Ono H.K., Shimizu S., Takahashi-Omoe H., Nakane A., Uchiyama T., Shinagawa K., Imanishi K. (2013). Emetic potentials of newly identified staphylococcal enterotoxin-like toxins. Infect. Immun..

[14] Thomas D.Y., Jarraud S., Lemercier B., Cozon G., Echasserieau K., Etienne J., Gougeon M.L., Lina G., Vandenesch F. (2006). Staphylococcal enterotoxin-like toxins U2 and V, two new staphylococcal superantigens arising from recombination within the enterotoxin gene cluster. Infect. Immun..

[15] Wilson G.J., Seo K.S., Cartwright R.A., Connelley T., Chuang-Smith O.N., Merriman J.A., Guinane C.M., Park J.Y., Bohach G.A., Schlievert P.M. (2011). A novel core genome-encoded superantigen contributes to lethality of community-associated MRSA necrotizing pneumonia. PLoS Pathog..

[16] Lina G., Bohach G.A., Nair S.P., Hiramatsu K., Jouvin-Marche E., Mariuzza R. (2004). Standard nomenclature for the superantigens expressed by Staphylococcus. J. Infect. Dis..

[17] Ono H.K., Hirose S., Narita K., Sugiyama M., Asano K., Hu D.L., Nakane A. (2019). Histamine release from intestinal mast cells induced by staphylococcal enterotoxin A (SEA) evokes vomiting reflex in common marmoset. PLoS Pathog..

[18] Umeda K., Ono H.K., Wada T., Motooka D., Nakamura H., Hu D.L. (2021). High production of egc2-related staphylococcal enterotoxins caused a food poisoning outbreak. Int. J. Food Microbiol..

[19] Sato’o Y., Omoe K., Naito I., Ono H.K., Nakane A., Sugai M., Yamagishi N., Hu D.L. (2014). Molecular epidemiology and identification of a *Staphylococcus aureus* clone causing food poisoning outbreaks in Japan. J. Clin. Microbiol..

[20] Alibayov B., Zdenkova K., Sykorova H., Demnerova K. (2014). Molecular analysis of *Staphylococcus aureus* pathogenicity islands (SaPI) and their superantigens combination of food samples. J. Microbiol. Methods.

[21] Sato’o Y., Omoe K., Ono H.K., Nakane A., Hu D.L. (2013). A novel comprehensive analysis method for *Staphylococcus aureus* pathogenicity islands. Microbiol. Immunol..

[22] Tallent S.M., Langston T.B., Moran R.G., Christie G.E. (2007). Transducing particles of *Staphylococcus aureus* pathogenicity island SaPI1 are comprised of helper phage-encoded proteins. J. Bacteriol..

[23] Novick R.P., Subedi A. (2007). The SaPIs: Mobile pathogenicity islands of Staphylococcus. Chem. Immunol. Allergy.

[24] Letertre C., Perelle S., Dilasser F., Fach P. (2003). Identification of a new putative enterotoxin SEU encoded by the *egc* cluster of *Staphylococcus aureus*. J. Appl. Microbiol..

[25] Monday S.R., Bohach G.A. (2001). Genes encoding staphylococcal enterotoxins G and I are linked and separated by DNA related to other staphylococcal enterotoxins. J. Nat. Toxins.

[26] Collery M.M., Smyth D.S., Tumilty J.J.G., Twohig J.M., Smyth C.J. (2009). Associations between enterotoxin gene cluster types egc1, egc2 and egc3, agr types, enterotoxin and enterotoxin-like gene profiles, and molecular typing characteristics of human nasal carriage and animal isolates of *Staphylococcus aureus*. J. Med. Microbiol..

[27] Omoe K., Hu D.L., Takahashi-Omoe H., Nakane A., Shinagawa K. (2005). Comprehensive analysis of classical and newly described staphylococcal superantigenic toxin genes in *Staphylococcus aureus* isolates. FEMS Microbiol. Lett..

[28] Betley M.J., Mekalanos J.J. (1985). Staphylococcal enterotoxin A is encoded by phage. Science.

[29] Soltis M.T., Mekalanos J.J., Betley M.J. (1990). Identification of a bacteriophage containing a silent staphylococcal variant enterotoxin gene (sezA+). Infect. Immun..

[30] Coleman D.C., Sullivan D.J., Russell R.J., Arbuthnott J.P., Carey B.F., Pomeroy H.M. (1989). *Staphylococcus aureus* bacteriophages mediating the simultaneous lysogenic conversion of beta-lysin, staphylokinase and enterotoxin A: Molecular mechanism of triple conversion. J. Gen. Microbiol..

[31] Zeaki N., Susilo Y.B., Pregiel A., Radstrom P., Schelin J. (2015). Prophage-encoded staphylococcal enterotoxin A: Regulation of production in *Staphylococcus aureus* strains representing different sea regions. Toxins.

[32] Couch J.L., Soltis M.T., Betley M.J. (1988). Cloning and nucleotide sequence of the type E staphylococcal enterotoxin gene. J. Bacteriol..

[33] Spaulding A.R., Salgado-Pabon W., Kohler P.L., Horswill A.R., Leung D.Y., Schlievert P.M. (2013). Staphylococcal and streptococcal superantigen exotoxins. Clin. Microbiol. Rev..

[34] Gunther S., Varma A.K., Moza B., Kasper K.J., Wyatt A.W., Zhu P., Rahman A.K.M.N., Li Y.L., Mariuzza R.A., McCormick J.K. (2007). A novel loop domain in superantigens extends their T cell receptor recognition site. J. Mol. Biol..

[35] Petersson K., Thunnissen M., Forsberg G., Walse B. (2002). Crystal structure of a SEA variant in complex with MHC class II reveals the ability of SEA to crosslink MHC molecules. Structure.

[36] Mollick J.A., Chintagumpala M., Cook R.G., Rich R.R. (1991). Staphylococcal exotoxin activation of T cells. Role of exotoxin-MHC class II binding affinity and class II isotype. J. Immunol..

[37] Herman A., Croteau G., Sekaly R.P., Kappler J., Marrack P. (1990). HLA-DR alleles differ in their ability to present staphylococcal enterotoxins to T cells. J. Exp. Med..

[38] Ferry T., Thomas D., Perpoint T., Lina G., Monneret G., Mohammedi I., Chidiac C., Peyramond D., Vandenesch F., Etienne J. (2008). Analysis of superantigenic toxin Vbeta T-cell signatures produced during cases of staphylococcal toxic shock syndrome and septic shock. Clin. Microbiol. Infect..

[39] Marrack P., Kappler J. (1990). The staphylococcal enterotoxins and their relatives. Science.

[40] Faulkner L., Cooper A., Fantino C., Altmann D.M., Sriskandan S. (2005). The mechanism of superantigen-mediated toxic shock: Not a simple Th1 cytokine storm. J. Immunol..

[41] Leder L., Llera A., Lavoie P.M., Lebedeva M.I., Hongmin Li H., Sékaly R.P., Bohach G.A., Gahr P.J., Schlievert P.M., Karjalainen K. (1998). A mutational analysis of the binding of staphylococcal enterotoxins B and C3 to the T cell receptor beta chain and major histocompatibility complex class II. J. Exp. Med..

[42] Li H., Llera A., Tsuchiya D., Leder L., Ysern X., Schlievert P.M., Karjalainen K., Mariuzza R.A. (1998). Three-dimensional structure of the complex between a T cell receptor beta chain and the superantigen staphylococcal enterotoxin B. Immunity.

[43] Ono H.K., Hirose S., Naito I., Sato’o Y., Asano K., Hu D.L., Omoe K., Nakane A. (2017). The emetic activity of staphylococcal enterotoxins, SEK, SEL, SEM, SEN and SEO in a small emetic animal model, the house musk shrew. Microbiol. Immunol..

[44] Swaminathan S., Furey W., Pletcher J., Sax M. (1992). Crystal structure of staphylococcal enterotoxin B, a superantigen. Nature.

[45] Swaminathan S., Furey W., Pletcher J., Sax M. (1995). Residues defining V beta specificity in staphylococcal enterotoxins. Nat. Struct. Biol..

[46] Swaminathan S., Yang D.S., Furey W., Abrams L., Pletcher J., Sax M. (1988). Crystallization and preliminary X-ray study of staphylococcal enterotoxin B. J. Mol. Biol..

[47] Li S.J., Hu D.L., Maina E.K., Shinagawa K., Omoe K., Nakane A. (2011). Superantigenic activity of toxic shock syndrome toxin-1 is resistant to heating and digestive enzymes. J. Appl. Microbiol..

[48] Hu D.L., Ono H.K., Isayama S., Okada R., Okamura M., Lei L.C., Liu Z.S., Zhang X.C., Liu M.Y., Cui J.C. (2017). Biological characteristics of staphylococcal enterotoxin Q and its potential risk for food poisoning. J. Appl. Microbiol..

[49] Jupin C., Anderson S., Damais C., Alouf J.E., Parant M. (1988). Toxic shock syndrome toxin 1 as an inducer of human tumor necrosis factors and gamma interferon. J. Exp. Med..

[50] Trede N.S., Geha R.S., Chatila T. (1991). Transcriptional activation of IL-1 beta and tumor necrosis factor-alpha genes by MHC class II ligands. J. Immunol..

[51] Tessier P.A., Naccache P.H., Diener K.R., Gladue R.P., Neote K.S., Clark-Lewis I., McColl S.R. (1998). Induction of acute inflammation in vivo by staphylococcal superantigens. II. Critical role for chemokines, ICAM-1, and TNF-alpha. J. Immunol..

[52] Irwin M.J., Hudson K.R., Fraser J.D., Gascoigne N.R. (1992). Enterotoxin residues determining T-cell receptor V beta binding specificity. Nature.

[53] Irwin M.J., Hudson K.R., Ames K.T., Fraser J.D., Gascoigne N.R. (1993). T-cell receptor beta-chain binding to enterotoxin superantigens. Immunol. Rev..

[54] Hu D.L., Cui J.C., Omoe K., Sashinami H., Yokomizo Y., Shinagawa K., Nakane A. (2005). A mutant of staphylococcal enterotoxin C devoid of bacterial superantigenic activity elicits a Th2 immune response for protection against *Staphylococcus aureus* infection. Infect. Immun..

[55] Hu D.L., Zhu G., Mori F., Omoe K., Okada M., Wakabayashi K., Kaneko S., Shinagawa K., Nakane A. (2007). Staphylococcal enterotoxin induces emesis through increasing serotonin release in intestine and it is downregulated by cannabinoid receptor 1. Cell. Microbiol..

[56] Hu D.L., Omoe K., Sashinami H., Shinagawa K., Nakane A. (2009). Immunization with a nontoxic mutant of staphylococcal enterotoxin A, SEAD227A, protects against enterotoxin-induced emesis in house musk shrews. J. Infect. Dis..

[57] Schad E.M., Zaitseva I., Zaitsev V.N., Dohlsten M., Kalland T., Schlievert P.M., Ohlendorf D.H., Svensson L.A. (1995). Crystal structure of the superantigen staphylococcal enterotoxin type A. EMBO J..

[58] Hu D.L., Omoe K., Nakane A., Sugii S., Ono K., Sasaki S., Shinagawa K. (1999). Studies on the functional site on staphylococcal enterotoxin A responsible for production of murine gamma interferon. FEMS Immunol. Med. Microbiol..

[59] Krakauer T. (2019). Staphylococcal Superantigens: Pyrogenic Toxins Induce Toxic Shock. Toxins.

[60] Omoe K., Nunomura W., Kato H., Li Z.J., Igarashi O., Araake M., Sano K., Ono H.K., Abe Y., Hu D.L. (2010). High affinity of interaction between superantigen and T cell receptor Vβ molecules induces a high level and prolonged expansion of superantigen-reactive CD4^+^ T cells. J. Biol. Chem..

[61] Suzuki Y., Omoe K., Hu D.L., Sato’o Y., Ono H.K., Monma C., Arai T., Konishi N., Kato R., Hirai A. (2014). Molecular epidemiological characterization of *Staphylococcus aureus* isolates originating from food poisoning outbreaks that occurred in Tokyo, Japan. Microbiol. Immunol..

[62] Chai S.J., Gu W., O’Connor K.A., Richardson L.C., Tauxe R.V. (2019). Incubation periods of enteric illnesses in foodborne outbreaks, United States, 1998-2013. Epidemiol. Infect..

[63] Tauxe R.V. (2002). Emerging foodborne pathogens. Int. J. Food Microbiol..

[64] Wieneke A.A., Roberts D., Gilbert R.J. (1993). Staphylococcal food poisoning in the United Kingdom, 1969–1990. Epidemiol. Infect..

[65] Veras J.F., do Carmo L.S., Tong L.C., Shupp J.W., Cummings C., dos Santos D.A., Cerqueira M.M.O.P., Cantini A., Nicoli J.R., Jett M. (2008). A study of the enterotoxigenicity of coagulase-negative and coagulase-positive staphylococcal isolates from food poisoning outbreaks in Minas Gerais, Brazil. Int. J. Infect. Dis..

[66] Kitamoto M., Kito K., Niimi Y., Shoda S., Takamura A., Hiramatsu T., Akashi T., Yokoi Y., Hirano H., Hosokawa M. (2009). Food poisoning by *Staphylococcus aureus* at a university festival. Jpn. J. Infect. Dis..

[67] Le Loir Y., Baron F., Gautier M. (2003). *Staphylococcus aureus* and food poisoning. Genet. Mol. Res..

[68] Hu D.L., Omoe K., Shimoda Y., Nakane A., Shinagawa K. (2003). Induction of emetic response to staphylococcal enterotoxins in the house musk shrew (*Suncus murinus*). Infect. Immun..

[69] Hu D.L., Omoe K., Saleh M.H., Ono K., Sugii S., Nakane A., Shinagawa K. (2001). Analysis of the epitopes on staphylococcal enterotoxin A responsible for emetic activity. J. Vet. Med. Sci..

[70] Omoe K., Imanishi K., Hu D.L., Kato H., Fugane Y., Abe Y., Hamaoka S., Watanabe Y., Nakane A., Uchiyama T. (2005). Characterization of novel staphylococcal enterotoxin-like toxin type P. Infect. Immun..

[71] Omoe K., Imanishi K., Hu D.L., Kato H., Takahashi-Omoe H., Nakane A., Uchiyama T., Shinagawa K. (2004). Biological properties of staphylococcal enterotoxin-like toxin type R. Infect. Immun..

[72] Ono H.K., Nishizawa M., Yamamoto Y., Hu D.L., Nakane A., Shinagawa K., Omoe K. (2012). Submucosal mast cells in the gastrointestinal tract are a target of staphylococcal enterotoxin type A. FEMS Immunol. Med. Microbiol..

[73] Hirose S., Ono H.K., Omoe K., Hu D.L., Asano K., Yamamoto Y., Nakane A. (2016). Goblet cells are involved in translocation of staphylococcal enterotoxin A in the intestinal tissue of house musk shrew (*Suncus murinus*). J. Appl. Microbiol..

[74] Todd J., Fishaut M., Kapral F., Welch T. (1978). Toxic-shock syndrome associated with phage-group-I Staphylococci. Lancet.

[75] Lindsay J.A., Ruzin A., Ross H.F., Kurepina N., Novick R.P. (1998). The gene for toxic shock toxin is carried by a family of mobile pathogenicity islands in *Staphylococcus aureus*. Mol. Microbiol..

[76] McCormick J.K., Yarwood J.M., Schlievert P.M. (2001). Toxic shock syndrome and bacterial superantigens: An update. Annu. Rev. Microbiol..

[77] Kim J., Urban R.G., Strominger J.L., Wiley D.C. (1994). Toxic shock syndrome toxin-1 complexed with a class II major histocompatibility molecule HLA-DR1. Science.

[78] Cui J.C., Zhang B.J., Lin Y.C., Wang Q.K., Qian A.D., Nakane A., Hu D.L., Tong G.Z. (2010). Protective effect of glutathione S-transferase-fused mutant staphylococcal enterotoxin C against *Staphylococcus aureus*-induced bovine mastitis. Vet. Immunol. Immunopathol..

[79] Shopsin B., Gomez M., Montgomery S.O., Smith D.H., Waddington M., Dodge D.E., Bost D.A., Riehman M., Naidich S., Kreiswirth B.N. (1999). Evaluation of protein A gene polymorphic region DNA sequencing for typing of *Staphylococcus aureus* strains. J. Clin. Microbiol..

[80] Becker S., Frankel M.B., Schneewind O., Missiakas D. (2014). Release of protein A from the cell wall of *Staphylococcus aureus*. Proc. Natl. Acad. Sci. USA.

[81] Kim H.K., Thammavongsa V., Schneewind O., Missiakas D. (2012). Recurrent infections and immune evasion strategies of *Staphylococcus aureus*. Curr. Opin. Microbiol..

[82] Guss B., Uhlen M., Nilsson B., Lindberg M., Sjoquist J., Sjodahl J. (1984). Region X, the cell-wall-attachment part of staphylococcal protein A. Eur. J. Biochem..

[83] Peterson P.K., Verhoef J., Sabath L.D., Quie P.G. (1977). Effect of protein A on staphylococcal opsonization. Infect. Immun..

[84] Graille M., Stura E.A., Corper A.L., Sutton B.J., Taussig M.J., Charbonnier J.B., Silverman G.J. (2000). Crystal structure of a *Staphylococcus aureus* protein A domain complexed with the Fab fragment of a human IgM antibody: Structural basis for recognition of B-cell receptors and superantigen activity. Proc. Natl. Acad. Sci. USA.

[85] Silverman G.J., Goodyear C.S. (2006). Confounding B-cell defences: Lessons from a staphylococcal superantigen. Nat. Rev. Immunol..

[86] Dumont A.L., Nygaard T.K., Watkins R.L., Smith A., Kozhaya L., Kreiswirth B.N., Shopsin B., Unutmaz D., Voyich J.M., Torres V.J. (2011). Characterization of a new cytotoxin that contributes to *Staphylococcus aureus* pathogenesis. Mol. Microbiol..

[87] Sun Y., Emolo C., Holtfreter S., Wiles S., Kreiswirth B., Missiakas D., Schneewind O. (2018). Staphylococcal protein A contributes to persistent colonization of mice with *Staphylococcus aureus*. J. Bacteriol..

[88] Kennedy A.D., Wardenburg B.J., Gardner D.J., Long D., Whitney A.R., Braughton K.R., Schneewind O., DeLeo F.R. (2010). Targeting of alpha-hemolysin by active or passive immunization decreases severity of USA300 skin infection in a mouse model. J. Infect. Dis..

[89] Kielian T., Cheung A., Hickey W.F. (2001). Diminished virulence of an alpha-toxin mutant of *Staphylococcus aureus* in experimental brain abscesses. Infect. Immun..

[90] O’Callaghan R.J., Callegan M.C., Moreau J.M., Green L.C., Foster T.J., Hartford O.M., Engel L.S., Hill J.M. (1997). Specific roles of alpha-toxin and beta-toxin during *Staphylococcus aureus* corneal infection. Infect. Immun..

[91] Suttorp N., Seeger W., Dewein E., Bhakdi S., Roka L. (1985). Staphylococcal alpha-toxin-induced PGI2 production in endothelial cells: Role of calcium. Am. J. Physiol..

[92] Bhakdi S., Muhly M., Korom S., Hugo F. (1989). Release of interleukin-1 beta associated with potent cytocidal action of staphylococcal alpha-toxin on human monocytes. Infect. Immun..

[93] Song L., Hobaugh M.R., Shustak C., Cheley S., Bayley H., Gouaux J.E. (1996). Structure of staphylococcal alpha-hemolysin, a heptameric transmembrane pore. Science.

[94] Freer J.H., Arbuthnott J.P., Bernheimer A.W. (1968). Interaction of staphylococcal alpha-toxin with artificial and natural membranes. J. Bacteriol..

[95] Watanabe M., Tomita T., Yasuda T. (1987). Membrane-damaging action of staphylococcal alpha-toxin on phospholipid-cholesterol liposomes. Biochim. Biophys. Acta.

[96] Valeva A., Hellmann N., Walev I., Strand D., Plate M., Boukhallouk F., Brack A., Hanada K., Decker H., Bhakdi S. (2006). Evidence that clustered phosphocholine head groups serve as sites for binding and assembly of an oligomeric protein pore. J. Biol. Chem..

[97] Wilke G.A., Wardenburg J.B. (2010). Role of a disintegrin and metalloprotease 10 in *Staphylococcus aureus* alpha-hemolysin-mediated cellular injury. Proc. Natl. Acad. Sci. USA.

[98] Popov L.M., Marceau C.D., Starkl P.M., Lumb J.H., Shah J., Guerrera D., Cooper R.L., Merakou C., Bouley D.M., Meng W. (2015). The adherens junctions control susceptibility to *Staphylococcus aureus* alpha-toxin. Proc. Natl. Acad. Sci. USA.

[99] Winter S.V., Zychlinsky A., Bardoel B.W. (2016). Genome-wide CRISPR screen reveals novel host factors required for *Staphylococcus aureus* alpha-hemolysin-mediated toxicity. Sci. Rep..

[100] von Hoven G., Rivas A.J., Neukirch C., Klein S., Hamm C., Qin Q., Meyenburg M., Füser S., Saftig P., Hellmann N. (2016). Dissecting the role of ADAM10 as a mediator of *Staphylococcus aureus* alpha-toxin action. Biochem. J..

[101] Lemjabbar H., Basbaum C. (2002). Platelet-activating factor receptor and ADAM10 mediate responses to *Staphylococcus aureus* in epithelial cells. Nat. Med..

[102] Maretzky T., Reiss K., Ludwig A., Buchholz J., Scholz F., Proksch E., de Strooper B., Hartmann D., Saftig P. (2005). ADAM10 mediates E-cadherin shedding and regulates epithelial cell-cell adhesion, migration, and beta-catenin translocation. Proc. Natl. Acad. Sci. USA.

[103] Schulz B., Pruessmeyer J., Maretzky T., Ludwig A., Blobel C.P., Saftig P., Reiss K. (2008). ADAM10 regulates endothelial permeability and T-Cell transmigration by proteolysis of vascular endothelial cadherin. Circ. Res..

[104] Colciaghi F., Borroni B., Pastorino L., Marcello E., Zimmermann M., Cattabeni F., Padovani A., Luca M.D. (2002). [alpha]-Secretase ADAM10 as well as [alpha]APPs is reduced in platelets and CSF of Alzheimer disease patients. Mol. Med..

[105] Hattori M., Osterfield M., Flanagan J.G. (2000). Regulated cleavage of a contact-mediated axon repellent. Science.

[106] Alonzo F., Torres V.J. (2014). The bicomponent pore-forming leucocidins of *Staphylococcus aureus*. Microbiol. Mol. Biol. Rev..

[107] Sugawara-Tomita N., Tomita T., Kamio Y. (2002). Stochastic assembly of two-component staphylococcal gamma-hemolysin into heteroheptameric transmembrane pores with alternate subunit arrangements in ratios of 3:4 and 4:3. J. Bacteriol..

[108] Bhakdi S., Tranum-Jensen J. (1991). Alpha-toxin of *Staphylococcus aureus*. Microbiol. Rev..

[109] Powers M.E., Kim H.K., Wang Y., Wardenburg J.B. (2012). ADAM10 mediates vascular injury induced by *Staphylococcus aureus* alpha-hemolysin. J. Infect. Dis..

[110] Bhakdi S., Muhly M., Mannhardt U., Hugo F., Klapettek K., Mueller-Eckhardt C., Roka L. (1988). Staphylococcal alpha toxin promotes blood coagulation via attack on human platelets. J. Exp. Med..

[111] Nygaard T.K., Pallister K.B., DuMont A.L., DeWald M., Watkins R.L., Pallister E.Q., Malone C., Griffith S., Horswill A.R., Torres V.J. (2012). Alpha-toxin induces programmed cell death of human T cells, B cells, and monocytes during USA300 infection. PLoS ONE.

[112] Wardenburg J.B., Patel R.J., Schneewind O. (2007). Surface proteins and exotoxins are required for the pathogenesis of *Staphylococcus aureus* pneumonia. Infect. Immun..

[113] Parker M.W., Feil S.C. (2005). Pore-forming protein toxins: From structure to function. Prog. Biophys. Mol. Biol..

[114] Berube B.J., Wardenburg J.B. (2013). *Staphylococcus aureus* alpha-toxin: Nearly a century of intrigue. Toxins.

[115] Lizak M., Yarovinsky T.O. (2012). Phospholipid scramblase 1 mediates type i interferon-induced protection against staphylococcal alpha-toxin. Cell Host Microbe.

[116] Grimminger F., Rose F., Sibelius U., Pötzsch B., Spriestersbach R., Bhakdi S., Suttorp N., Seeger W. (1997). Human endothelial cell activation and mediator release in response to the bacterial exotoxins *Escherichia coli* hemolysin and staphylococcal alpha-toxin. J. Immunol..

[117] Rose F., Dahlem G., Guthmann B., Grimminger F., Maus U., Hänze J., Duemmer N., Grandel U., Seeger W., Ghofrani H.A. (2002). Mediator generation and signaling events in alveolar epithelial cells attacked by *S. aureus* alpha-toxin. Am. J. Physiol. Lung Cell. Mol. Physiol..

[118] Grumann D., Nubel U., Broker B.M. (2014). *Staphylococcus aureus* toxins—Their functions and genetics. Infect. Genet. Evol..

[119] Inoshima I., Inoshima N., Wilke G.A., Powers M.E., Frank K.M., Wang Y., Wardenburg J.B. (2011). A *Staphylococcus aureus* pore-forming toxin subverts the activity of ADAM10 to cause lethal infection in mice. Nat. Med..

[120] Kolata J., Bode L.G., Holtfreter S., Steil L., Kusch H., Holtfreter B., Albrecht D., Hecker M., Engelmann S., van Belkum A. (2011). Distinctive patterns in the human antibody response to *Staphylococcus aureus* bacteremia in carriers and non-carriers. Proteomics.

[121] Fritz S.A., Tiemann K.M., Hogan P.G., Epplin E.K., Rodriguez M., Al-Zubeidi D.N., Wardenburg J.B., Hunstad D.A. (2013). A serologic correlate of protective immunity against community-onset *Staphylococcus aureus* infection. Clin. Infect. Dis..

[122] Doery H.M., Magnusson B.J., Cheyne I.M., Sulasekharam J. (1963). A phospholipase in staphylococcal toxin which hydrolyses sphingomyelin. Nature.

[123] Huseby M., Shi K., Brown C.K., Digre J., Mengistu F., Seo K.S., Bohach G.A., Schlievert P.M., Ohlendorf D.H., Earhart C.A. (2007). Structure and biological activities of beta toxin from *Staphylococcus aureus*. J. Bacteriol..

[124] Huseby M.J., Kruse A.C., Digre J., Kohler P.L., Vocke J.A., Mann E.E., Bayles K.W., Bohach G.A., Schlievert P.M., Ohlendorf D.H. (2010). Beta toxin catalyzes formation of nucleoprotein matrix in staphylococcal biofilms. Proc. Natl. Acad. Sci. USA.

[125] Low D.K., Freer J.H., Arbuthnott J.P., Mollby R., Wadstrom T. (1974). Consequences of spingomyelin degradation in erythrocyte ghost membranes by staphylococcal beta-toxin (sphingomyelinase C). Toxicon.

[126] Cifrian E., Guidry A.J., Bramley A.J., Norcross N.L., Bastida-Corcuera F.D., Marquardt W.W. (1996). Effect of staphylococcal beta toxin on the cytotoxicity, proliferation and adherence of *Staphylococcus aureus* to bovine mammary epithelial cells. Vet. Microbiol..

[127] Katayama Y., Baba T., Sekine M., Fukuda M., Hiramatsu K. (2013). Beta-hemolysin promotes skin colonization by *Staphylococcus aureus*. J. Bacteriol..

[128] Tajima A., Iwase T., Shinji H., Seki K., Mizunoe Y. (2009). Inhibition of endothelial interleukin-8 production and neutrophil transmigration by *Staphylococcus aureus* beta-hemolysin. Infect. Immun..

[129] Salgado-Pabon W., Herrera A., Vu B.G., Stach C.S., Merriman J.A., Spaulding A.R., Schlievert P.M. (2014). *Staphylococcus aureus* beta-toxin production is common in strains with the beta-toxin gene inactivated by bacteriophage. J. Infect. Dis..

[130] Hayashida A., Bartlett A.H., Foster T.J., Park P.W. (2009). *Staphylococcus aureus* beta-toxin induces lung injury through syndecan-1. Am. J. Pathol..

[131] Yoong P., Torres V.J. (2013). The effects of *Staphylococcus aureus* leukotoxins on the host: Cell lysis and beyond. Curr. Opin. Microbiol..

[132] Prevost G., Cribier B., Couppie P., Petiau P., Supersac G., Finck-Barbançon V., Monteil H., Piemont Y. (1995). Panton-Valentine leucocidin and gamma-hemolysin from *Staphylococcus aureus* ATCC 49775 are encoded by distinct genetic loci and have different biological activities. Infect. Immun..

[133] Morinaga N., Kaihou Y., Noda M. (2003). Purification, cloning and characterization of variant LukE-LukD with strong leukocidal activity of staphylococcal bi-component leukotoxin family. Microbiol. Immunol..

[134] Yamashita K., Kawai Y., Tanaka Y., Hirano N., Kaneko J., Tomita N., Ohta M., Kamio Y., Yao M., Tanaka I. (2011). Crystal structure of the octameric pore of staphylococcal gamma-hemolysin reveals the beta-barrel pore formation mechanism by two components. Proc. Natl. Acad. Sci. USA.

[135] Aman M.J., Karauzum H., Bowden M.G., Nguyen T.L. (2010). Structural model of the pre-pore ring-like structure of Panton-Valentine leukocidin: Providing dimensionality to biophysical and mutational data. J. Biomol. Struct. Dyn..

[136] Woodin A.M. (1960). Purification of the two components of leucocidin from *Staphylococcus aureus*. Biochem. J..

[137] Spaan A.N., van Strijp J.A.G., Torres V.J. (2017). Leukocidins: Staphylococcal bi-component pore-forming toxins find their receptors. Nat. Rev. Microbiol..

[138] Yamashita D., Sugawara T., Takeshita M., Kaneko J., Kamio Y., Tanaka I., Tanaka Y., Yao M. (2014). Molecular basis of transmembrane beta-barrel formation of staphylococcal pore-forming toxins. Nat. Commun..

[139] Yanai M., Rocha M.A., Matolek A.Z., Chintalacharuvu A., Taira Y., Chintalacharuvu K., Beenhouwer D.O. (2014). Separately or combined, LukG/LukH is functionally unique compared to other staphylococcal bicomponent leukotoxins. PLoS ONE.

[140] Perret M., Badiou C., Lina G., Burbaud S., Benito Y., Bes M., Cottin V., Couzon F., Juruj C., Dauwalder O. (2012). Cross-talk between *Staphylococcus aureus* leukocidins-intoxicated macrophages and lung epithelial cells triggers chemokine secretion in an inflammasome-dependent manner. Cell. Microbiol..

[141] Holzinger D., Gieldon L., Mysore V., Nippe N., Taxman D.J., Duncan J.A., Broglie P.M., Marketon K., Austermann J., Vogl T. (2012). *Staphylococcus aureus* Panton-Valentine leukocidin induces an inflammatory response in human phagocytes via the NLRP3 inflammasome. J. Leukoc. Biol..

[142] Melehani J.H., James D.B., DuMont A.L., Torres V.J., Duncan J.A. (2015). *Staphylococcus aureus* Leukocidin A/B (LukAB) Kills Human Monocytes via Host NLRP3 and ASC when Extracellular, but Not Intracellular. PLoS Pathog..

[143] Vrieling M., Boerhout E.M., van Wigcheren G.F., Koymans K.J., Mols-Vorstermans T.G., de Haas C.J.C., Aerts P.C., Daemen I.J.J.M., van Kessel K.P.M., Koets A.P. (2016). LukMF’ is the major secreted leukocidin of bovine *Staphylococcus aureus* and is produced in vivo during bovine mastitis. Sci. Rep..

[144] Yamada T., Tochimaru N., Nakasuji S., Hata E., Kobayashi H., Eguchi M., Kaneko J., Kamio Y., Kaidoh T., Takeuchi S. (2005). Leukotoxin family genes in *Staphylococcus aureus* isolated from domestic animals and prevalence of lukM-lukF-PV genes by bacteriophages in bovine isolates. Vet. Microbiol..

[145] McCarthy A.J., Lindsay J.A. (2013). *Staphylococcus aureus* innate immune evasion is lineage-specific: A bioinfomatics study. Infect. Genet. Evol..

[146] von Eiff C., Friedrich A.W., Peters G., Becker K. (2004). Prevalence of genes encoding for members of the staphylococcal leukotoxin family among clinical isolates of *Staphylococcus aureus*. Diagn. Microbiol. Infect. Dis..

[147] Seals D.F., Courtneidge S.A. (2003). The ADAMs family of metalloproteases: Multidomain proteins with multiple functions. Genes Dev..

[148] Fackrell H.B., Wiseman G.M. (1976). Properties of the gamma haemolysin of *Staphylococcus aureus* ‘Smith 5R’. J. Gen. Microbiol..

[149] Spaan A.N., Reyes-Robles T., Badiou C., Cochet S., Boguslawski K.M., Yoong P., Day C.J., de Haas C.J.C., van Kessel K.P.M., Vandenesch F. (2015). *Staphylococcus aureus* Targets the Duffy Antigen Receptor for Chemokines (DARC) to Lyse Erythrocytes. Cell Host Microbe.

[150] Noda M., Hirayama T., Kato I., Matsuda F. (1980). Crystallization and properties of staphylococcal leukocidin. Biochim. Biophys. Acta.

[151] Spaan A.N., Vrieling M., Wallet P., Badiou C., Reyes-Robles T., Ohneck E.A., Benito Y., de Haas C.J.C., Day C.J., Jennings M.P. (2014). The staphylococcal toxins gamma-haemolysin AB and CB differentially target phagocytes by employing specific chemokine receptors. Nat. Commun..

[152] Hildebrand A., Pohl M., Bhakdi S. (1991). *Staphylococcus aureus* alpha-toxin. Dual mechanism of binding to target cells. J. Biol. Chem..

[153] DeLeo F.R., Kennedy A.D., Chen L., Wardenburg J.B., Kobayashi S.D., Mathema B., Braughton K.R., Whitney A.R., Villaruz A.E., Martens C.A. (2011). Molecular differentiation of historic phage-type 80/81 and contemporary epidemic *Staphylococcus aureus*. Proc. Natl. Acad. Sci. USA.

[154] Siqueira J.A., Speeg-Schatz C., Freitas F.I., Sahel J., Monteil H., Prevost G. (1997). Channel-forming leucotoxins from *Staphylococcus aureus* cause severe inflammatory reactions in a rabbit eye model. J. Med. Microbiol..

[155] Munoz-Planillo R., Franchi L., Miller L.S., Nunez G. (2009). A critical role for hemolysins and bacterial lipoproteins in *Staphylococcus aureus*-induced activation of the Nlrp3 inflammasome. J. Immunol..

[156] Nilsson I.M., Hartford O., Foster T., Tarkowski A. (1999). Alpha-toxin and gamma-toxin jointly promote *Staphylococcus aureus* virulence in murine septic arthritis. Infect. Immun..

[157] Malachowa N., Whitney A.R., Kobayashi S.D., Sturdevant D.E., Kennedy A.D., Braughton K.R., Shabb D.W., Diep B.A., Chambers H.F., Otto M. (2011). Global changes in *Staphylococcus aureus* gene expression in human blood. PLoS ONE.

[158] Goerke C., Pantucek R., Holtfreter S., Schulte B., Zink M., Grumann D., Bröker B.M., Doskar J., Wolz C. (2009). Diversity of prophages in dominant *Staphylococcus aureus* clonal lineages. J. Bacteriol..

[159] Kaneko J., Kimura T., Narita S., Tomita T., Kamio Y. (1998). Complete nucleotide sequence and molecular characterization of the temperate staphylococcal bacteriophage phiPVL carrying Panton-Valentine leukocidin genes. Gene.

[160] Spaan A.N., Henry T., van Rooijen W.J.M., Perret M., Badiou C., Aerts P.C., Kemmink J., de Haas C.J.C., van Kessel K.P.M., Vandenesch F. (2013). The staphylococcal toxin Panton-Valentine leukocidin targets human C5a receptors. Cell Host Microbe.

[161] Spaan A.N., Schiepers A., de Haas C.J., van Hooijdonk D.D.J.J., Badiou C., Contamin H., Vandenesch F., Lina G., Gerard N.P., Gerard C. (2015). Differential interaction of the staphylococcal toxins Panton-Valentine leukocidin and gamma-hemolysin CB with human C5a receptors. J. Immunol..

[162] Couppie P., Cribier B., Prevost G. (1994). Leukocidin from *Staphylococcus aureus* and cutaneous infections: An epidemiologic study. Arch. Dermatol..

[163] Prevost G., Couppie P., Prevost P., Gayet S., Petiau P., Cribier B., Monteil H., Piemont Y. (1995). Epidemiological data on *Staphylococcus aureus* strains producing synergohymenotropic toxins. J. Med. Microbiol..

[164] Diep B.A., Chan L., Tattevin P., Kajikawa O., Martin T.R., Basuino L., Mai T.T., Marbach H., Braughton K.R., Whitney A.R. (2010). Polymorphonuclear leukocytes mediate *Staphylococcus aureus* Panton-Valentine leukocidin-induced lung inflammation and injury. Proc. Natl. Acad. Sci. USA.

[165] Cremieux A.C., Dumitrescu O., Lina G., Vallee C., Côté J.F., Muffat-Joly M., Lilin T., Etienne J., Vandenesch F., Saleh-Mghir A. (2009). Panton-valentine leukocidin enhances the severity of community-associated methicillin-resistant *Staphylococcus aureus* rabbit osteomyelitis. PLoS ONE.

[166] Graves S.F., Kobayashi S.D., Braughton K.R., Whitney A.R., Sturdevant D.E., Rasmussen D.L., Kirpotina L.N., Quinn M.T., DeLeo F.R. (2012). Sublytic concentrations of *Staphylococcus aureus* Panton-Valentine leukocidin alter human PMN gene expression and enhance bactericidal capacity. J. Leukoc. Biol..

[167] Kobayashi S.D., Malachowa N., Whitney A.R., Braughton K.R., Gardner D.J., Long D., Wardenburg J.B., Schneewind O., Otto M., DeLeo F.R. (2011). Comparative analysis of USA300 virulence determinants in a rabbit model of skin and soft tissue infection. J. Infect. Dis..

[168] Chi C.Y., Lin C.C., Liao I.C., Yao Y.C., Shen F.C., Liu C.C., Lin C.F. (2014). Panton-Valentine leukocidin facilitates the escape of *Staphylococcus aureus* from human keratinocyte endosomes and induces apoptosis. J. Infect. Dis..

[169] Boakes E., Kearns A.M., Ganner M., Perry C., Hill R.L., Ellington M.J. (2011). Distinct bacteriophages encoding Panton-Valentine leukocidin (PVL) among international methicillin-resistant *Staphylococcus aureus* clones harboring PVL. J. Clin. Microbiol..

[170] Gillet Y., Issartel B., Vanhems P., Fournet J.C., Lina G., Bes M., Vandenesch P.F., Piémont Y., Brousse N., Floret P.D. (2002). Association between *Staphylococcus aureus* strains carrying gene for Panton-Valentine leukocidin and highly lethal necrotising pneumonia in young immunocompetent patients. Lancet.

[171] DuMont A.L., Yoong P., Liu X., Day J.C., Chumbler N.M., James D.B.A., Alonzo F., Bode N.J., Lacy D.B., Jennings M.P. (2014). Identification of a crucial residue required for *Staphylococcus aureus* LukAB cytotoxicity and receptor recognition. Infect. Immun..

[172] Badarau A., Rouha H., Malafa S., Logan D.T., Håkansson M., Stulik L., Dolezilkova I., Teubenbacher A., Gross K., Maierhofer B. (2015). Structure-function analysis of heterodimer formation, oligomerization, and receptor binding of the *Staphylococcus aureus* bi-component toxin LukGH. J. Biol. Chem..

[173] Ventura C.L., Malachowa N., Hammer C.H., Nardone G.A., Robinson M.A., Kobayashi S.D., DeLeo F.R. (2010). Identification of a novel *Staphylococcus aureus* two-component leukotoxin using cell surface proteomics. PLoS ONE.

[174] Malachowa N., Kobayashi S.D., Braughton K.R., Whitney A.R., Parnell M.J., Gardner D.J., DeLeo F.R. (2012). *Staphylococcus aureus* leukotoxin GH promotes inflammation. J. Infect. Dis..

[175] DuMont A.L., Yoong P., Day C.J., Alonzo F., McDonald W.H., Jennings M.P., Torres V.J. (2013). *Staphylococcus aureus* LukAB cytotoxin kills human neutrophils by targeting the CD11b subunit of the integrin Mac-1. Proc. Natl. Acad. Sci. USA.

[176] Dozois A., Thomsen I., Jimenez-Truque N., Soper N., Pearson A., Mohamed-Rambaran P., Dettorre K.B., Creech C.B., Wright S.W. (2015). Prevalence and molecular characteristics of methicillin-resistant *Staphylococcus aureus* among skin and soft tissue infections in an emergency department in Guyana. Emerg. Med. J..

[177] Thomsen I.P., Dumont A.L., James D.B., Yoong P., Saville B.R., Soper N., Torres V.J., Creech C.B. (2014). Children with invasive *Staphylococcus aureus* disease exhibit a potently neutralizing antibody response to the cytotoxin LukAB. Infect. Immun..

[178] Chadha A.D., Thomsen I.P., Jimenez-Truque N., Soper N.R., Jones L.S., Sokolow A.G., Torres V.J., Creech C.B. (2016). Host response to *Staphylococcus aureus* cytotoxins in children with cystic fibrosis. J. Cyst. Fibros..

[179] DuMont A.L., Yoong P., Surewaard B.G., Benson M.A., Nijland R., van Strijp J.A.G., Torres V.J. (2013). *Staphylococcus aureus* elaborates leukocidin AB to mediate escape from within human neutrophils. Infect. Immun..

[180] Gravet A., Colin D.A., Keller D., Girardot R., Monteil H., Prevost G. (1998). Characterization of a novel structural member, LukE-LukD, of the bi-component staphylococcal leucotoxins family. FEBS Lett..

[181] Liu C., Chen Z.J., Sun Z., Feng X., Zou M., Cao W., Wang S., Zeng J., Wang Y., Sun M. (2015). Molecular characteristics and virulence factors in methicillin-susceptible, resistant, and heterogeneous vancomycin-intermediate *Staphylococcus aureus* from central-southern China. J. Microbiol. Immunol. Infect..

[182] Haveri M., Roslof A., Rantala L., Pyorala S. (2007). Virulence genes of bovine *Staphylococcus aureus* from persistent and nonpersistent intramammary infections with different clinical characteristics. J. Appl. Microbiol..

[183] Alonzo F., Benson M.A., Chen J., Novick R.P., Shopsin B., Torres V.J. (2012). *Staphylococcus aureus* leucocidin ED contributes to systemic infection by targeting neutrophils and promoting bacterial growth in vivo. Mol. Microbiol..

[184] Reyes-Robles T., Alonzo F., Kozhaya L., Lacy D.B., Unutmaz D., Torres V.J. (2013). *Staphylococcus aureus* leukotoxin ED targets the chemokine receptors CXCR1 and CXCR2 to kill leukocytes and promote infection. Cell Host Microbe.

[185] Alonzo F., Kozhaya L., Rawlings S.A., Reyes-Robles T., DuMont A.L., Myszka D.G., Landau N.R., Unutmaz D., Torres V.J. (2013). CCR5 is a receptor for *Staphylococcus aureus* leukotoxin ED. Nature.

[186] Reyes-Robles T., Lubkin A., Alonzo F., Lacy D.B., Torres V.J. (2016). Exploiting dominant-negative toxins to combat *Staphylococcus aureus* pathogenesis. EMBO Rep..

[187] Fromageau A., Cunha P., Gilbert F.B., Rainard P. (2011). Purified *Staphylococcus aureus* leukotoxin LukM/F’ does not trigger inflammation in the bovine mammary gland. Microb. Pathog..

[188] Vrieling M., Koymans K.J., Heesterbeek D.A., Aerts P.C., Rutten V.P.M.G., de Haas C.J.C., van Kessel K.P.M., Koets A.P., Nijland R., van Strijp J.A.G. (2015). Bovine *Staphylococcus aureus* Secretes the Leukocidin LukMF’ To Kill Migrating Neutrophils through CCR1. mBio.

[189] Koop G., Vrieling M., Storisteanu D.M., Lok L.S.C., Monie T., van Wigcheren G., Raisen C., Ba X., Gleadall N., Hadjirin N. (2017). Identification of LukPQ, a novel, equid-adapted leukocidin of *Staphylococcus aureus*. Sci. Rep..

[190] Merriman J.A., Klingelhutz A.J., Diekema D.J., Leung D.Y., Schlievert P.M. (2015). Novel *Staphylococcus aureus* Secreted Protein Alters Keratinocyte Proliferation and Elicits a Proinflammatory Response In Vitro and In Vivo. Biochemistry.

[191] Mehlin C., Headley C.M., Klebanoff S.J. (1999). An inflammatory polypeptide complex from Staphylococcus epidermidis: Isolation and characterization. J. Exp. Med..

[192] Wang R., Braughton K.R., Kretschmer D., Bach T.H.L., Queck S.Y., Li M., Kennedy A.D., Dorward D.W., Klebanoff S.J., Peschel A. (2007). Identification of novel cytolytic peptides as key virulence determinants for community-associated MRSA. Nat. Med..

[193] Peschel A., Otto M. (2013). Phenol-soluble modulins and staphylococcal infection. Nat. Rev. Microbiol..

[194] Towle K.M., Lohans C.T., Miskolzie M., Acedo J.Z., van Belkum M.J., Vederas J.C. (2016). Solution Structures of Phenol-Soluble Modulins alpha1, alpha3, and beta2, Virulence Factors from *Staphylococcus aureus*. Biochemistry.

[195] Otto M. (2014). Phenol-soluble modulins. Int. J. Med. Microbiol..

[196] Chikara K., Yuki S., Mariko I., Yosuke O., Han M., Gentaro N., Tomoko F., Shunsuke N., Xiao H., Kazuaki O. (2013). Mobile genetic element SCC*mec*-encoded *psm-mec* RNA suppresses translation of *agr*A and attenuates MRSA virulence. PLoS Pathog..

[197] Rasigade J.P., Trouillet-Assant S., Ferry T., Diep B.A., Sapin A., Lhoste L., Ranfaing J., Badiou C., Benito Y., Bes M. (2013). PSMs of hypervirulent *Staphylococcus aureus* act as intracellular toxins that kill infected osteoblasts. PLoS ONE.

[198] Surewaard B.G., de Haas C.J., Vervoort F., Rigby K.M., DeLeo F.R., Otto M., van Strijp J.A.G., Nijland R. (2013). Staphylococcal alpha-phenol soluble modulins contribute to neutrophil lysis after phagocytosis. Cell. Microbiol..

[199] Kretschmer D., Gleske A.K., Rautenberg M., Wang R., Köberle M., Bohn E., Schöneberg T., Rabiet M.J., Boulay F., Klebanoff S.J. (2010). Human formyl peptide receptor 2 senses highly pathogenic *Staphylococcus aureus*. Cell Host Microbe.

[200] Periasamy S., Joo H.S., Duong A.C., Bach T.M.L., Tan V.Y., Chatterjee S.S., Cheung G.Y.C., Otto M. (2012). How *Staphylococcus aureus* biofilms develop their characteristic structure. Proc. Natl. Acad. Sci. USA.

[201] Yamaguchi T., Hayashi T., Takami H., Nakasone K., Ohnishi M., Nakayama K., Yamada S., Komatsuzawa H., Sugai M. (2000). Phage conversion of exfoliative toxin A production in *Staphylococcus aureus*. Mol. Microbiol..

[202] Yamaguchi T., Nishifuji K., Sasaki M., Fudaba Y., Aepfelbacher M., Takata T., Ohara M., Komatsuzawa H., Amagai M., Sugai M. (2002). Identification of the *Staphylococcus aureus etd* pathogenicity island which encodes a novel exfoliative toxin, ETD, and EDIN-B. Infect. Immun..

[203] Gismene C., Hernández González J.E., Santisteban A.R.N., Ziem Nascimento A.F., dos Santos Cunha L., de Moraes F.R., de Oliveira C.L.P., Oliveira C.C., Jocelan Scarin Provazzi P., Pascutti P.G. (2022). *Staphylococcus aureus* Exfoliative Toxin E, Oligomeric State and Flip of P186: Implications for Its Action Mechanism. Int. J. Mol. Sci..

[204] Bukowski M., Wladyka B., Dubin G. (2010). Exfoliative toxins of *Staphylococcus aureus*. Toxins.

[205] Vath G.M., Earhart C.A., Rago J.V., Kim M.H., Bohach G.A., Schlievert P.M., Ohlendorf G.H. (1997). The structure of the superantigen exfoliative toxin A suggests a novel regulation as a serine protease. Biochemistry.

[206] Amagai M., Yamaguchi T., Hanakawa Y., Nishifuji K., Sugai M., Stanley J.R. (2002). Staphylococcal exfoliative toxin B specifically cleaves desmoglein 1. J. Investig. Dermatol..

[207] Ladhani S. (2001). Recent developments in staphylococcal scalded skin syndrome. Clin. Microbiol. Infect..

[208] Amagai M., Matsuyoshi N., Wang Z.H., Andl C., Stanley J.R. (2000). Toxin in bullous impetigo and staphylococcal scalded-skin syndrome targets desmoglein 1. Nat. Med..

[209] Cribier B., Piemont Y., Grosshans E. (1994). Staphylococcal scalded skin syndrome in adults: A clinical review illustrated with a new case. J. Am. Acad. Dermatol..

[210] Melish M.E., Glasgow L.A. (1970). The staphylococcal scalded-skin syndrome. N. Engl. J. Med..

